# Edge-rich Cu-N_3_ single atom nanozyme drives lipid switching to potentiate tumor catalytic therapy

**DOI:** 10.7150/thno.128026

**Published:** 2026-04-08

**Authors:** Xin Xing, Jiaji Yu, Yajie Zhang, Xue Chen, Liang Chen, Meng Du, Jie Ding, Zhiyi Chen, Li Li, Junjie Cheng

**Affiliations:** 1 Department of Stomatology, The First Affiliated Hospital of Wannan Medical College (Yijishan Hospital of Wannan Medical College), Wuhu 241001, China.; 2 Department of Nutrition and Food Hygiene, School of Public Health; Department of Radiology, Zhongda Hospital, Nurturing Center of Jiangsu Province for State Laboratory of AI Imaging & Interventional Radiology, School of Medicine; Southeast University, Nanjing 210009, China.; 3 Central Laboratory, Department of Biobank, Nanjing Hospital of Chinese Medicine Affiliated to Nanjing University of Chinese Medicine, Nanjing 210022, China.; 4 Key Laboratory of Medical Imaging Precision Theranostics and Radiation Protection, College of Hunan Province, Hengyang Medical School, The Affiliated Changsha Central Hospital, University of South China, Changsha, China.; 5 Max Planck Institute of Microstructure Physics, Weinberg 2, Halle, 06120, Germany.

**Keywords:** nanozyme, single atom catalyst, catalytic therapy, ferroptosis, tumor therapy

## Abstract

**Rationale:**

Tumor catalytic therapy represents a promising antitumor approach by inducing ferroptosis and overcoming apoptosis-related resistance mechanisms. Its efficacy is primarily dictated by the extent of membrane lipid peroxidation (LPO). However, tumor cells may evade ferroptosis through metabolic reprogramming that enriches monounsaturated fatty acids (MUFAs) in membrane lipids, thereby diminishing oxidative vulnerability. Hence, strategies that simultaneously enhance catalytic ROS production and reprogram lipid metabolism are required to address this challenge.

**Methods:**

To overcome this limitation, a novel Cu-N_3_ single-atom nanozyme with edge enrichment (*ER*-Cu_1_SAZyme) was developed, characterized by a hollow porous structure and catalytically active sites concentrated along the edges. This design optimizes atom utilization, increases local electronic density, and lowers the reaction energy barrier, thereby promoting potent intracellular reactive oxygen species (ROS) generation. To further sensitize tumors to ferroptosis, *ER*-Cu_1_SAZyme was combined with sirolimus (Srl), an FDA-approved drug, to create the Srl/*ER*-Cu_1_SAZyme nanomedicine for coordinated catalytic and metabolic regulation.

**Results:**

The Srl/*ER*-Cu_1_SAZyme formulation simultaneously inhibits stearoyl-CoA desaturase 1 (SCD1)-mediated MUFA synthesis and upregulates ACSL4, thereby shifting the membrane lipid composition toward a ferroptosis-sensitive phenotype and enhancing nanozyme-induced LPO. This dual catalytic-metabolic strategy increases ferroptosis susceptibility while reducing metastatic potential linked to excessive membrane fluidity. In tumor-bearing mouse models, Srl/*ER*-Cu_1_SAZyme treatment led to a 33-fold reduction in tumor volume compared to the untreated group, without observable systemic toxicity.

**Conclusions:**

These results highlight the effectiveness of integrating edge-enriched single-atom catalysis with lipid metabolic modulation to enhance ferroptosis-based tumor therapy. The Srl/*ER*-Cu_1_SAZyme nanomedicine offers a safe and highly potent approach for dual catalytic-metabolic regulation in cancer treatment.

## Introduction

Nanozymes are synthetic nanomaterials that mimic the catalytic functions of natural enzymes, gaining increasing attention in tumor therapy 1-4. By catalyzing redox reactions and facilitating substrate conversions *in vivo* 5-7, nanozymes have emerged as promising agents for tumor catalytic treatment, particularly in inducing ferroptosis 8, 9. Ferroptosis is a regulated form of cell death triggered by excessive oxidative stress and the accumulation of lipid peroxides (LPO), which leads to irreversible membrane damage 10. This mechanism offers an effective alternative for eliminating tumor cells resistant to apoptosis-based therapies 11-14. However, many tumor cells acquire adaptive resistance to oxidative damage by reprogramming lipid metabolism, typically by increasing the proportion of monounsaturated fatty acids (MUFAs) in membrane lipids 15-18. The single double bond in MUFAs imparts high oxidative stability, protecting membrane integrity and suppressing lipid peroxidation (LPO), thereby reducing sensitivity to ferroptosis. Correcting this metabolic shift, by reducing MUFA levels and restoring the ratio of polyunsaturated fatty acids (PUFAs) to MUFAs, has thus become a promising strategy to enhance ferroptosis and improve therapeutic outcomes.

Recent developments in nanozyme engineering have significantly enhanced catalytic performance by optimizing the structural precision and atomic distribution of active sites 19. Among these, single-atom nanozymes (SAZymes) represent a new generation of catalytic materials, offering maximized atomic efficiency and well-defined coordination environments. These properties enable superior catalytic activity and selectivity compared to conventional nanoparticle-based systems 20. However, despite these advances, the catalytic efficiency of many SAzymes remains limited by suboptimal atomic utilization and electronic structure 21. Most conventional SAzymes exhibit in-plane M-N_4_ coordination, where only a fraction of catalytic atoms are accessible for substrate interaction, and the local electronic configuration is not ideally tuned for reactive oxygen species (ROS) generation 22. To overcome these limitations, developing single-atom nanozymes with abundant accessible active sites and optimized atomic coordination is crucial. Such advancements would amplify oxidative stress within the tumor microenvironment (TME) while disrupting metabolic pathways that facilitate membrane remodeling.

**Scheme 1:**
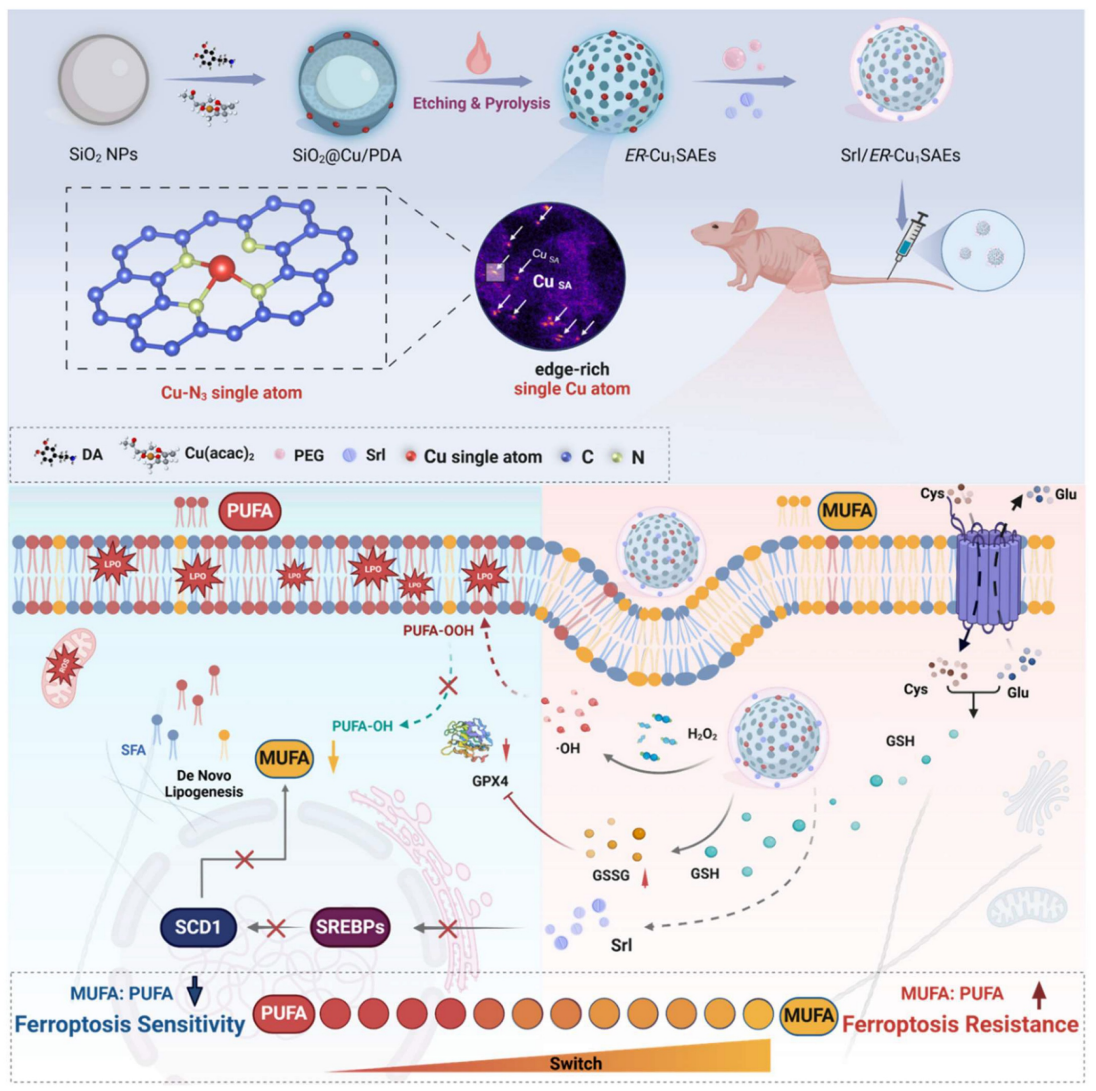
Schematic illustration of Srl/*ER*-Cu_1_SAZyme in tumor catalytic therapy. The edge-enriched Cu-N_3_ single-atom nanozyme (*ER*-Cu_1_SAZyme) induces intense oxidative stress in tumor cells by depleting intracellular glutathione (GSH) and catalyzing the conversion of hydrogen peroxide (H_2_O_2_) into reactive hydroxyl radicals (•OH), triggering oxidative damage. Moreover, the FDA-approved sirolimus (Srl) effectively inhibits MUFA synthesis by downregulating SREBP1 and SCD1 activity, thereby restoring the MUFA/PUFA ratio in membrane lipids and rendering tumor cells more susceptible to LPO and ferroptosis.

In this study, a novel edge-enriched Cu-N_3_ single-atom nanozyme (*ER*-Cu_1_SAZyme) was designed to meet the dual requirements of enhanced catalytic efficiency and metabolic regulation. Unlike conventional planar SAzymes, *ER*-Cu_1_SAZyme features a hollow porous nitrogen-doped carbon framework with Cu atoms preferentially anchored at edge sites, forming abundant Cu-N_3_ coordination centers. This unique edge-enriched architecture increases atomic utilization, boosts local electronic density, and reduces the reaction energy barrier, thereby facilitating the efficient conversion of H_2_O_2_ into highly reactive hydroxyl radicals (•OH) and depleting intracellular glutathione (GSH). The resultant ROS surge disrupts redox balance, triggers extensive LPO, and ultimately induces ferroptosis. Additionally, the hollow, porous structure of *ER*-Cu_1_SAZyme serves as an ideal platform for loading and delivering small molecules, including clinical drugs. To sensitize tumors to ferroptosis, the FDA-approved drug sirolimus (Srl) was incorporated into *ER*-Cu_1_SAZyme, forming the Srl/*ER*-Cu_1_SAZyme nanomedicine. Srl inhibits stearoyl-CoA desaturase 1 (SCD1)-mediated MUFA biosynthesis while promoting ACSL4-mediated PUFA utilization, thereby shifting membrane lipid composition toward a ferroptosis-susceptible phenotype. This synergistic combination amplifies ferroptosis sensitivity while mitigating the excessive membrane fluidity and metastatic potential often associated with PUFA supplementation. As illustrated in Scheme 1, Srl/*ER*-Cu_1_SAZyme integrates single-atom catalytic ROS generation with metabolic modulation of membrane lipids, offering a robust and safe ferroptosis-based antitumor strategy. This approach presents a promising solution and a new opportunity to conjugate nanocatalysts with clinical drugs for more effective tumor treatment.

## Results and Discussion

### Preparation and characterization of the edge-rich single-Cu-atom SAZyme (*ER*-Cu_1_SAZyme)

**Figure 1:**
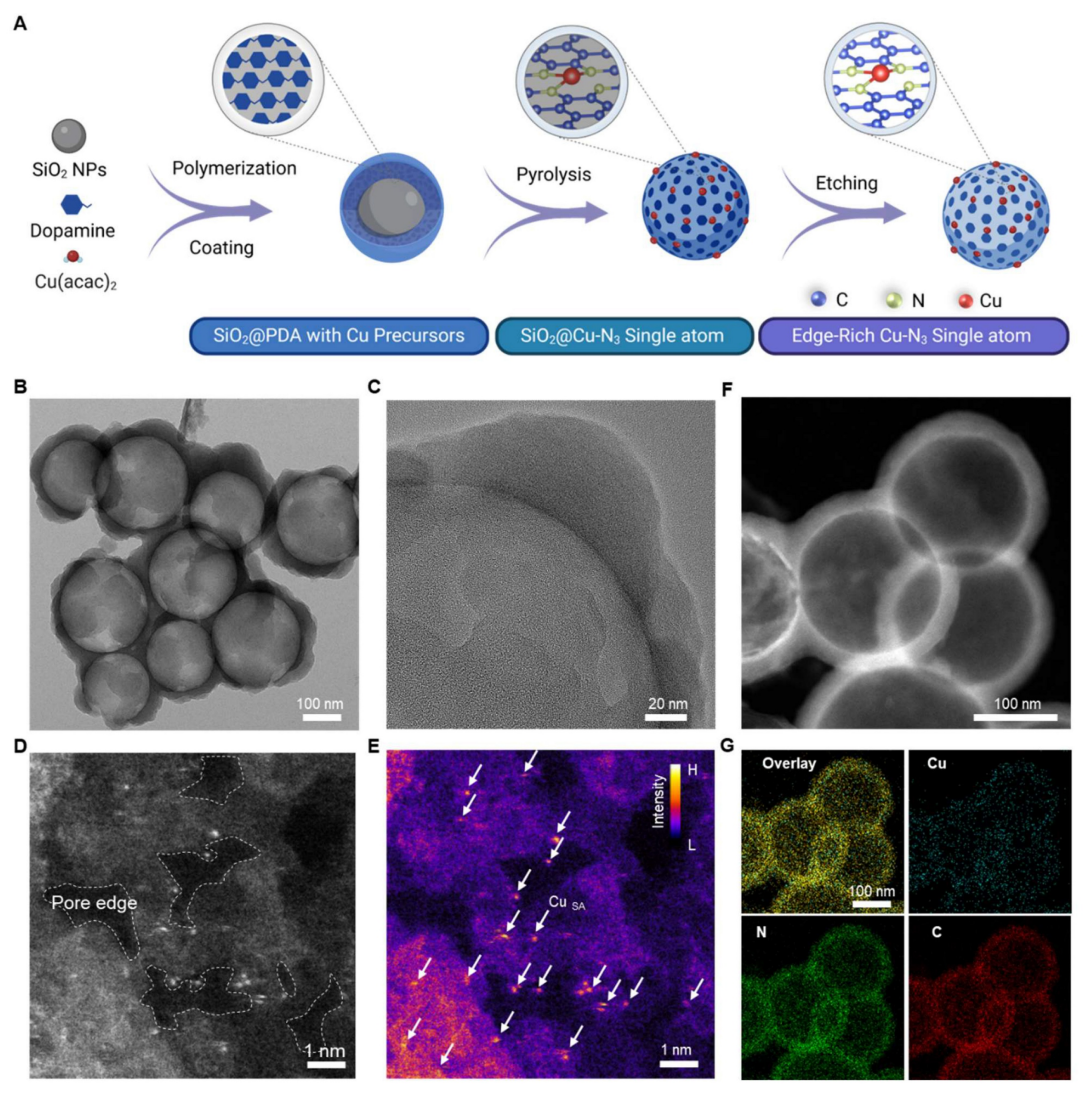
Synthesis and structural characterizations of the *ER*-Cu_1_SAZyme. (A) Illustration of the preparation process of *ER*-Cu_1_SAZyme *via* a thermal anchoring strategy. (B-C) TEM images of *ER*-Cu_1_SAZyme. (D-E) Atomic-resolution HAADF-STEM image and corresponding intensity maps with color profile. (F) HAADF-STEM image. (G) Corresponding energy-dispersive X-ray elemental mapping, indicating the homogeneous distribution of copper (Cu), nitrogen (N), and carbon (C) in *ER*-Cu_1_SAZyme.

Copper (Cu) atoms are key cofactors in biological enzymatic reactions due to their redox flexibility and biocompatibility 23. Additionally, Cu offers a cost-effective alternative to noble metals such as platinum (Pt), palladium (Pd), and gold (Au), making it an attractive candidate for single-atom nanozyme design 24. To optimize Cu catalytic efficiency within the TME, a novel hollow porous structure was engineered to serve as the substrate for the nanozyme, providing abundant edge sites to anchor Cu catalytic centers. The synthesis of *ER*-Cu_1_SAZyme follows a three-step process, as illustrated in Figure 1A. First, mesoporous silica (SiO_2_) was synthesized using the sol-gel method as a hard template. Dopamine and a copper precursor were introduced to initiate *in situ* polymerization, forming a continuous polydopamine (PDA) coating on the SiO_2_ surface. Following high-temperature pyrolysis, the PDA coating converted into a nitrogen-doped carbon (CN) framework with atomically dispersed Cu species. Finally, the SiO_2_ core was selectively removed using NaOH, resulting in a hollow structure with a porous CN shell. This architecture significantly increases the surface area and exposes edge sites, providing an ideal scaffold for stabilizing Cu single atoms.

**Figure 2:**
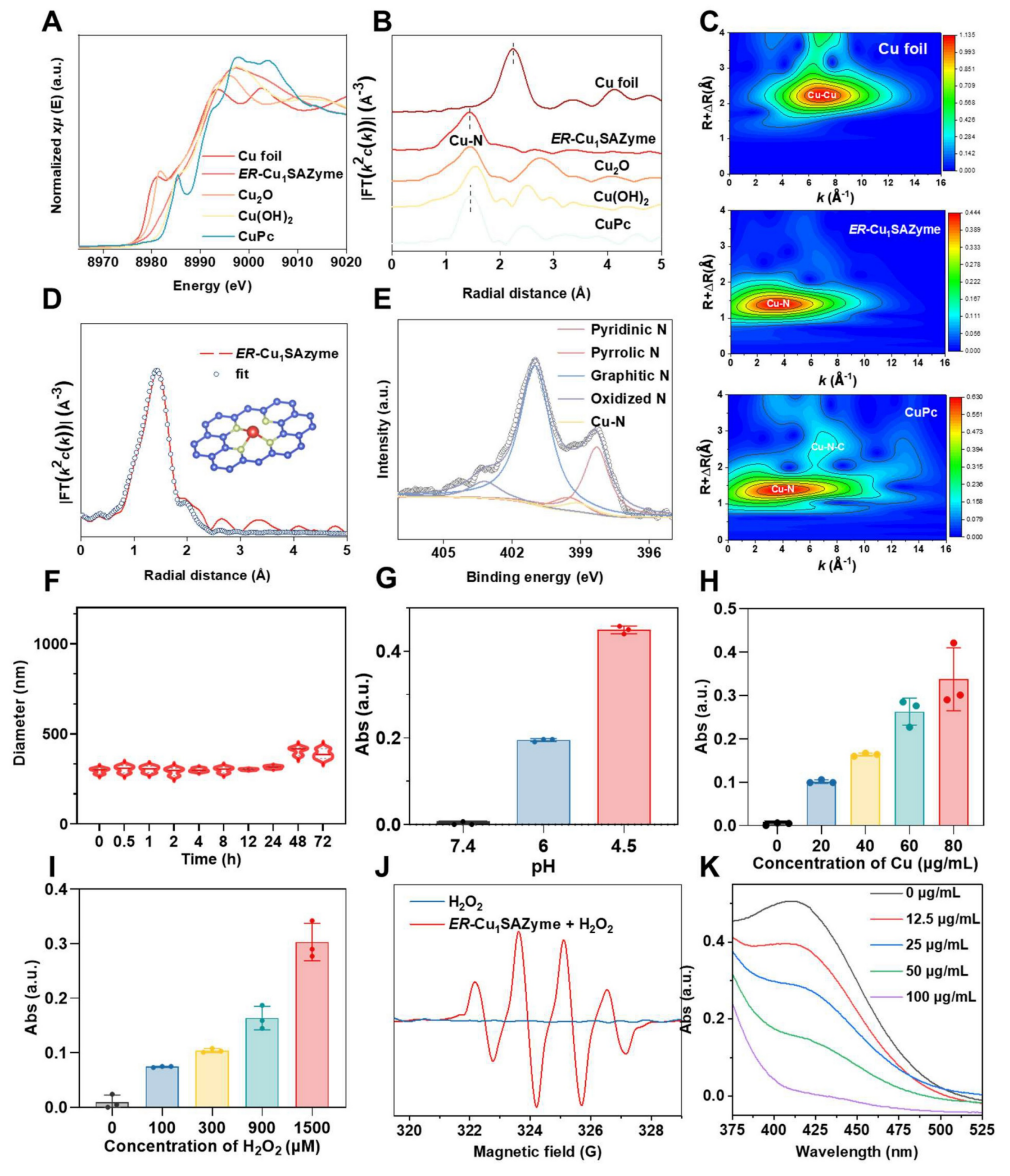
Atomic structural and catalytic characterizations of *ER*-Cu_1_SAZyme. (A) Cu K-edge XANES spectra of *ER*-Cu_1_SAZyme and reference materials. (B) Fourier-transformed EXAFS signal of *ER*-Cu_1_SAZyme and references. (C) EXAFS wavelet-transformed curves of Cu foil, *ER*-Cu_1_SAZyme, and CuPc. (D) EXAFS fitting of *ER*-Cu_1_SAZyme. (E) N 1s XPS spectra of *ER*-Cu_1_SAZyme. (F) Stability analysis of the nanomedicine. (G-I) POD-like activity of *ER*-Cu_1_SAZyme in TMB chromogenic reaction at pH 7.4, 6.0, and 4.5 (G); varying *ER*-Cu_1_SAZyme concentrations from 0 to 80 μg/mL (H); and varying H_2_O_2_ concentrations from 0 to 1500 μM (I). (J) ESR spectra for •OH detection using DMPO as a trapping agent. (K) GSH depletion activity of *ER*-Cu_1_SAZyme *via* the DTNB reduction assay.

The *ER*-Cu_1_SAZyme was extensively characterized using multiple techniques. Transmission electron microscopy (TEM) revealed a well-defined hollow spherical morphology with an average diameter of approximately 250 nm (Figures 1B and S1), and a porous shell thickness ranging from 20 to 40 nm (Figure 1C). To further confirm the hollow and porous structure of *ER*-Cu_1_SAZyme, Brunauer-Emmett-Teller (BET) surface area analysis was conducted. The N_2_ adsorption-desorption isotherms and corresponding pore-size distribution are shown (Figure S2). These results reveal a mesoporous structure with a high surface area of 1376 m^2^ g^-1^, complementing the TEM and HAADF-STEM observations. This design promotes the uniform dispersion and stable coordination of single Cu atoms, particularly at the edge sites, ensuring maximal surface exposure. The increased surface area and enhanced accessibility of active sites contribute to the improved catalytic efficiency of the SAZyme. Atomic-resolution high-angle annular dark-field scanning transmission electron microscopy (HAADF-STEM) provided detailed insight into the distribution of Cu atoms on the *ER*-Cu_1_SAZyme. Discrete Cu atoms were clearly observed as bright spots, highlighted within white circles (Figure 1D). In contrast to the widely reported in-plane single-atom catalysts with Cu-N_4_ atomic structures, the synthesized *ER*-Cu_1_SAZyme features a hollow CN framework with abundant pores, with the active Cu species predominantly located at the edges of these pores. Accordingly, color-contrast images further highlight the edge-localized dispersion of single Cu atoms on the porous CN framework, clearly verifying their effective dispersion throughout the structure (Figure 1E). Additionally, inductively coupled plasma mass spectrometry (ICP-MS) analysis revealed that the Cu content in *ER*-Cu_1_SAZyme is approximately 0.6% (w/w), further confirming the presence of copper species (Table S1). The PEGylated nanomedicine exhibited a hydrated particle size of 300 nm with a zeta potential of +6 mV (Figure S3). The particle size remained stable for over 24 hours, with only a slight increase observed at 48 hours and stabilization by 72 hours, indicating good colloidal stability and excellent dispersibility, making it suitable for *in vivo* applications (Figure 2F).

### Atomic coordination characterizations of the *ER*-Cu_1_SAZyme

Synchrotron-based X-ray absorption spectroscopy (XAS) was employed to investigate the chemical state and local atomic environment of Cu in *ER*-Cu_1_SAZyme. The Cu K-edge X-ray absorption near-edge structure (XANES) spectra revealed that the absorption edge of *ER*-Cu_1_SAZyme shifted to higher energy compared to Cu foil, indicating that the Cu in *ER*-Cu_1_SAZyme is in an oxidized state (Figure 2A). By comparing with reference materials such as Cu foil, Cu_2_O, Cu(OH)_2_, and CuPc, the average Cu valence in *ER*-Cu_1_SAZyme was found to lie between +1 and +2, as the absorption edge of *ER*-Cu_1_SAZyme falls between those of Cu_2_O and Cu(OH)_2_ (Figure S4) 25. Fourier transform extended X-ray absorption fine structure (EXAFS) analysis provided further insights into the local coordination of Cu. Unlike Cu foil, which exhibited a characteristic Cu-Cu peak around 2.2 Å in R-space, *ER*-Cu_1_SAZyme displayed a prominent Cu-N peak at approximately 1.4 Å, confirming the isolated nature of Cu atoms and their predominant Cu-N coordination (Figures 2B and S5). Additionally, wavelet transform (WT) EXAFS analysis revealed strong Cu-N coordination at ~1.4 Å in both *ER*-Cu_1_SAZyme and CuPc, whereas Cu foil showed strong Cu-Cu coordination at ~2.2 Å (Figure 2C). These results further supported the isolated atomic dispersion of Cu in *ER*-Cu_1_SAZyme.

EXAFS fitting suggested a Cu-N coordination number of 3.12 for *ER*-Cu_1_SAZyme (Figure 2D and Table S2), confirming the presence of a Cu-N_3_ structure in the first coordination shell. Furthermore, X-ray photoelectron spectroscopy (XPS) validated the interaction between Cu, N, and C. The N 1s spectrum showed characteristic peaks corresponding to pyridinic N at ~398.5 eV, Cu-N coordination at ~399.1 eV, pyrrolic N at ~399.6 eV, graphitic N at ~401.1 eV, and oxidized N at ~403.1 eV, consistent with previously reported Cu-N-C single-atom systems (Figure 2E) 26, 27. The presence of pyridinic and pyrrolic nitrogen confirmed that isolated Cu is coordinated with approximately three pyridinic N atoms, predominantly forming the Cu-N_3_ structure in *ER*-Cu_1_SAZyme.

Despite the low Cu content, characteristic peaks for Cu 2p_3/2_ and Cu 2p_1/2_ were detected at 934.7 eV and 954.6 eV, respectively, corresponding to Cu^2+^ species, while peaks at 932.7 eV and 952.4 eV were attributed to Cu^+^ species. A satellite peak at 944.4 eV further confirmed the oxidation state of copper in *ER*-Cu_1_SAZyme (Figure S6) 28. The Cu content determined by XPS was 1.15% by mass, which is higher than the value obtained from energy-dispersive X-ray spectroscopy (EDS) analysis (0.73%), indicating that Cu species are more enriched on the surface of the hollow NC structure (Table S1). This observation aligns with the surface-sensitive nature of XPS, which captures surface-loaded species with greater precision than bulk analysis techniques like EDS 6. Additionally, graphitic and oxidized nitrogen enhance the conductivity and catalytic stability of the nanomaterial. The C 1s spectrum showed three distinct peaks at 290.0 eV, 287.9 eV, 286.1 eV, and 284.8 eV, corresponding to C=O, C-O, C-N, and C=C bonds, respectively (Figure S7) 29. These results validate the successful fabrication of *ER*-Cu_1_SAZyme, with atomically dispersed Cu sites supported by the CN framework, a configuration expected to yield excellent catalytic performance based on its structural and compositional attributes.

To improve the dispersibility and biocompatibility of *ER*-Cu_1_SAZyme under physiological conditions, nanoparticles were coated with 1,2-distearoyl-sn-glycero-3-phosphoethanolamine-polyethylene glycol (DSPE-PEG). In this process, the hydrophobic DSPE segment preferentially adsorbs onto the nanozyme surface, while the hydrophilic PEG chains extend outward into the aqueous environment, forming a monolayer on the nanoparticle surface 30-32. Simultaneously, Srl was loaded *via* hydrophobic interactions, forming a stable therapeutic nanomedicine (Srl/*ER*-Cu_1_SAZyme). As the feeding ratio of Srl increased, the amount of drug loaded onto *ER*-Cu_1_SAZyme increased accordingly, though encapsulation efficiency began to decline once the ratio exceeded 10:1. Therefore, a 10:1 feeding ratio was selected for subsequent experiments (Figure S8). The PEGylated nanomedicine remained stable in physiological mimicking conditions for over 24 hours, with only a slight increase in size observed after 48 hours, stabilizing at 72 hours, indicating excellent dispersibility for potential *in vivo* applications (Figure 2F).

### The catalytic performance of *ER*-Cu_1_SAZyme

The catalytic activity of *ER*-Cu_1_SAZyme was evaluated through its peroxidase (POD)-like activity using the 3,3',5,5'-tetramethylbenzidine (TMB) colorimetric assay. *ER*-Cu_1_SAZyme effectively catalyzed the conversion of H_2_O_2_ into highly reactive hydroxyl radicals (•OH), leading to the rapid oxidation of the TMB reagent to its blue oxidized form, which exhibited a characteristic absorption peak at 652 nm, correlating with concentration. As shown in Figure 2G, no significant absorbance signal was observed at pH 7.4, but a notable increase in absorbance occurred at lower pH levels, indicating the ability of the SAZyme to catalyze ROS production specifically within the acidic TME. To further assess catalytic performance under TME-simulating conditions, the catalytic activity was closely monitored at pH 6. Figure 2H illustrates a gradual increase in absorbance with higher concentrations of *ER*-Cu_1_SAZyme, suggesting that elevated SAZyme concentrations enhance the catalytic reaction. Similar concentration-dependent results were observed with the TMB (Figure S9). Moreover, as the concentration of H_2_O_2_ increased, absorbance also increased, demonstrating that the ROS generation reaction is more effective in the TME, where H_2_O_2_ levels are typically higher compared to normal tissues, thus enhancing tumor-targeting capabilities (Figure 2I).

Kinetic analyses were conducted using both the Michaelis-Menten and Lineweaver-Burk models. The calculated Km and Vmax values were 2.06 mM and 0.13 μM·s^-1^ for the Michaelis-Menten model, and 1.92 mM and 0.13 μM·s^-1^ for the Lineweaver-Burk model, respectively (Figures S10-11). These results indicate that *ER*-Cu_1_SAZyme exhibits significantly higher catalytic activity than a planar non-edge Cu-N_4_ single-atom counterpart, which was prepared by lowering the pyrolysis temperature to suppress edge-site formation. Despite having a similar elemental composition, this non-edge control sample lacks abundant low-coordinated edge sites, resulting in a planar coordination environment (Figures S12-13). Furthermore, *ER*-Cu_1_SAZyme demonstrates superior performance compared to several previously reported Cu-based single-atom nanozymes 33-36, confirming the effectiveness of the edge-enriched structural design. Additionally, the catalase (CAT), superoxide dismutase (SOD), and glutathione peroxidase (GPX)-like activities of *ER*-Cu_1_SAZyme were examined to confirm its selective catalytic properties. Results showed only weak CAT-like activity, while SOD- and GPX-like activities were barely measurable (Figures S14-S16). This selective catalytic behavior ensures stable and predictable catalytic performance, which is advantageous for controlled ROS generation and maintaining therapeutic precision in tumor treatment.

To identify the ROS generated by *ER*-Cu_1_SAZyme, electron spin resonance (ESR) analysis was conducted using 5,5-dimethyl-1-pyrroline N-oxide (DMPO) as a spin-trapping agent. The resulting 1:2:2:1 peak pattern confirmed the presence of •OH, indicating that *ER*-Cu_1_SAZyme acts as a POD mimic by catalyzing the conversion of H_2_O_2_ into •OH in tumor tissue (Figure 2J). Furthermore, the GSH-depleting activity of *ER*-Cu_1_SAZyme was assessed using the 5,5'-dithiobis (2-nitrobenzoic acid) (DTNB) reduction assay. The decrease in absorbance of the yellow DTNB indicates GSH oxidation in a reaction catalyzed by *ER*-Cu_1_SAZyme (Figure 2K). These findings highlight the exceptional ability of *ER*-Cu_1_SAZyme to generate highly reactive •OH radicals while simultaneously depleting key endogenous antioxidants like GSH. This dual action facilitates lipid oxidation in cellular membranes, making *ER*-Cu_1_SAZyme a potent agent for inducing oxidative stress in tumor cells.

The catalytic performance of *ER*-Cu_1_SAZyme before and after DSPE-PEG modification was also evaluated. The modified *ER*-Cu_1_SAZyme retained its efficient POD-like catalytic activity under physiological conditions, with only a slight decrease in activity compared to the unmodified nanozyme. This reduction may be due to partial blockage of catalytic sites or limited substrate diffusion (Figure S17).

### DFT studies on the activity of *ER*-Cu_1_SAZyme

**Figure 3:**
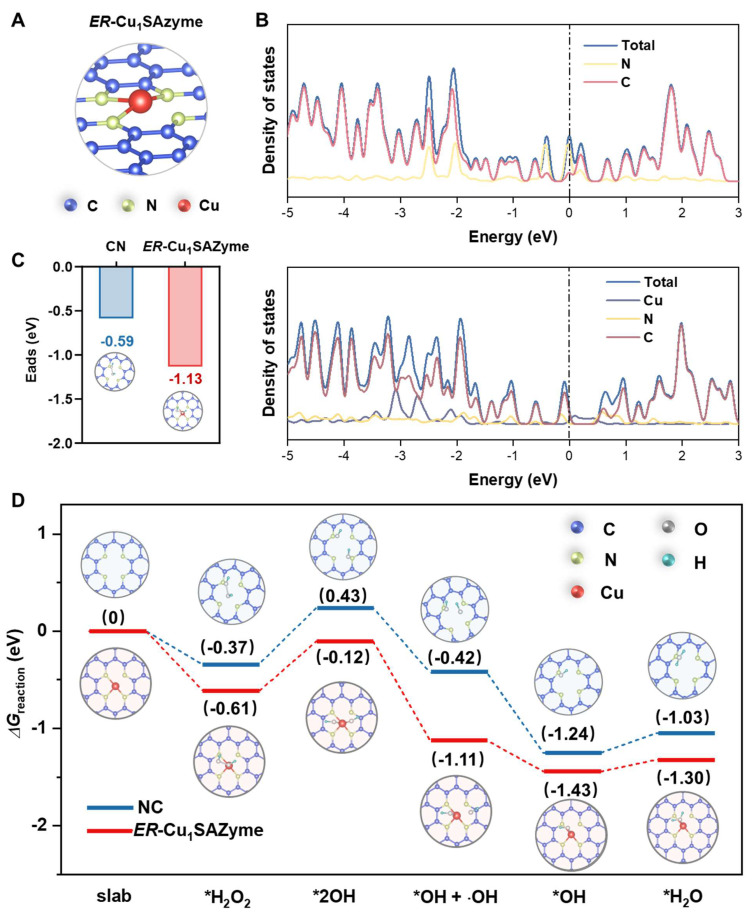
DFT studies on the activity of *ER*-Cu_1_SAZyme. (A) The coordination structure of *ER*-Cu_1_SAZyme. (B) The density of states of CN and *ER*-Cu_1_SAZyme. (C) Adsorption energy contrast for H_2_O_2_ molecule adsorption on NC and *ER*-Cu_1_SAZyme. (D) Energy profile diagram illustrating the possible reaction paths for H_2_O_2_ activation over the catalyst.

To gain deeper insight into the remarkable catalytic performance of *ER*-Cu_1_SAZyme in generating •OH radicals, density functional theory (DFT) calculations were performed to investigate the conversion process of H_2_O_2_ during the POD-like catalytic reaction. Initially, the coordination structure of *ER*-Cu_1_SAZyme was confirmed through EXAFS data fitting, revealing that Cu atoms are coordinated with an average of 3.12 nitrogen atoms at a path distance of about 1.92 Å (Table S2). This confirms that the primary coordination structure of *ER*-Cu_1_SAZyme is Cu-N_3_ (Figures 3A and S18). Following this, two catalyst models were constructed for analysis: *ER*-Cu_1_SAZyme and a control model lacking the Cu active center, referred to as NC. The density of states (DOS) analysis revealed distinct differences between the CN substrate and the Cu-containing model (Figure 3B). In the CN substrate without Cu, the DOS is primarily influenced by C states, with minimal contributions from N states. In contrast, the inclusion of Cu significantly alters the DOS, particularly in the energy range near the Fermi level (0 eV). The Cu atom introduces states around -3 eV, -2 eV, and just below the Fermi level, indicating strong interactions between the Cu atom and the surrounding CN framework. These interactions enhance the electron transport capacity of the SAZyme. Additionally, the increased complexity of the DOS near the Fermi level suggests that the presence of Cu creates new electronic states and modifies the distribution of existing ones, which is essential for the efficient decomposition of H_2_O_2_ during POD-like reactions.

The catalytic performance of *ER*-Cu_1_SAZyme is further supported by its stronger interaction with H_2_O_2_ compared to the CN substrate, as evidenced by the lower adsorption energy (-1.13 eV vs. -0.59 eV). This indicates that *ER*-Cu_1_SAZyme forms a more stable bond with H_2_O_2_, which enhances its catalytic activity. The energy profile (Figure 3D) outlines the entire catalytic process, beginning with the adsorption of H_2_O_2_ onto the catalyst. *ER*-Cu_1_SAZyme demonstrates lower reaction free energy, reflecting a higher adsorption affinity that facilitates subsequent steps. The adsorbed H_2_O_2_ dissociates into two *OH intermediates, with one desorbing to release an •OH radical. The remaining *OH reacts with hydrogen to form *H_2_O, and finally, the catalyst returns to its original state after H_2_O desorption. Throughout this process, *ER*-Cu_1_SAZyme consistently exhibits lower free energy than the CN substrate, confirming that the inclusion of Cu active centers significantly enhances the reaction efficiency, making *ER*-Cu_1_SAZyme an effective POD catalyst.

### The catalytic performance of Srl/*ER*-Cu_1_SAZyme in tumor cells

**Figure 4:**
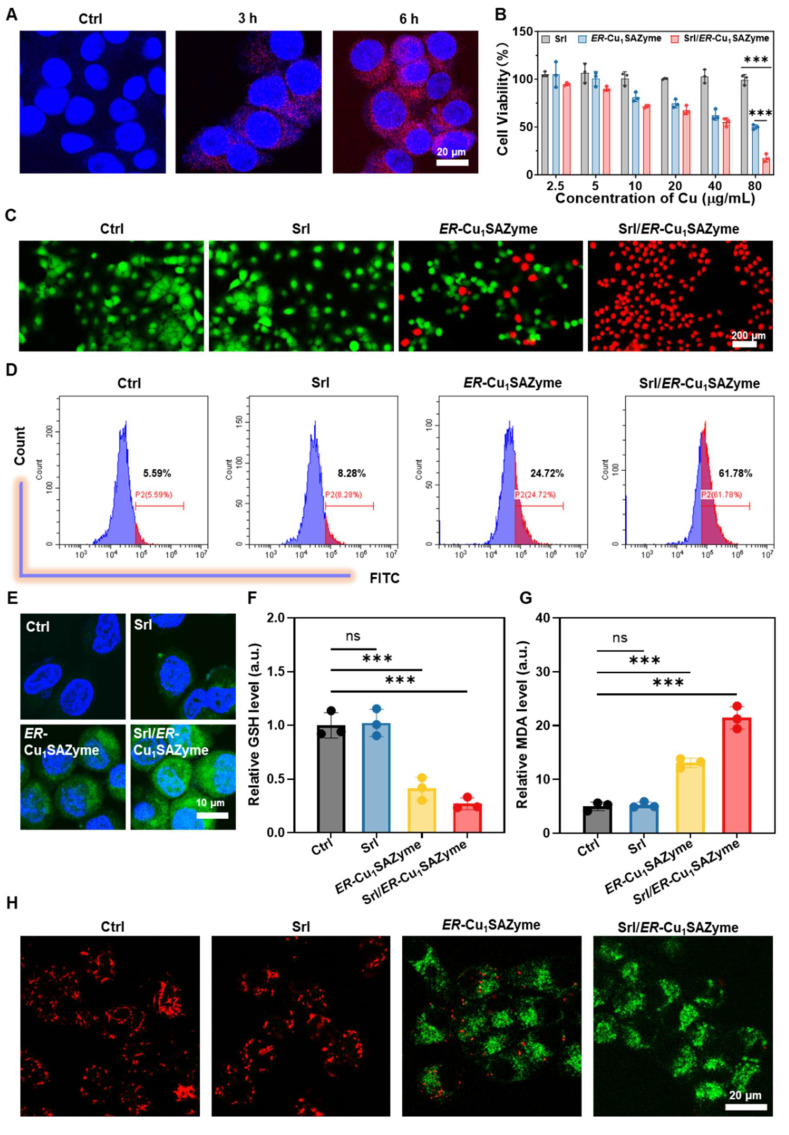
*In vitro* catalytic performance of *ER*-Cu_1_SAZyme. (A) Confocal microscopy images of Cal-27 cells after incubation with RF/*ER*-Cu_1_SAZyme (red: RF/*ER*-Cu_1_SAZyme, blue: Hoechst). (B) Cell viability of Cal-27 cells after treatment with Srl/*ER*-Cu_1_SAZyme for 48 hours. (C) Live/dead cell assay showing co-staining with Calcein-AM (green for live cells) and PI (red for dead cells). (D-E) Analysis of ROS levels in Cal-27 cells using DCFH-DA staining by flow cytometry (D) and confocal laser scanning microscopy (CLSM) (E). (F) Intracellular GSH levels in Cal-27 cells after incubation with Srl/*ER*-Cu_1_SAZyme. (G) Intracellular MDA levels in Cal-27 cells after incubation with Srl/*ER*-Cu_1_SAZyme. (H) CLSM images analyzing mitochondrial depolarization using a JC-1 kit. Data are presented as mean ± s.d., with all measurements (n) being biologically independent. Two-group comparisons were performed using a two-tailed unpaired Student's t-test, and multiple comparisons were analyzed by one-way ANOVA with Tukey's post hoc test.

Oral squamous cell carcinoma (OSCC) is an aggressive malignancy associated with dysregulated lipid metabolism, which drives tumor progression and therapeutic resistance 37, 38. In this study, OSCC Cal-27 cells were used to evaluate the therapeutic efficacy of Srl/*ER*-Cu_1_SAZyme. To assess the uptake efficiency of the SAZymes, red fluorescent-labeled *ER*-Cu_1_SAZyme (RF/*ER*-Cu_1_SAZyme) was incubated with the cells. The results showed that RF/*ER*-Cu_1_SAZyme was effectively internalized by Cal-27 cells in a time-dependent manner, laying the groundwork for subsequent therapeutic applications (Figures 4A and S19).

Following confirmation of cellular uptake, the cytotoxic effects of Srl/*ER*-Cu_1_SAZyme on Cal-27 cells were assessed using a CCK-8 assay. Treatment with *ER*-Cu_1_SAZyme significantly decreased the viability of Cal-27 cells, and the reduction was further pronounced when combined with Srl (Srl/*ER*-Cu_1_SAZyme) (Figure 4B). At a concentration of 80 μg/mL, the survival rate of cells treated with *ER*-Cu_1_SAZyme was approximately 50%, while the Srl/*ER*-Cu_1_SAZyme group exhibited a dramatic decrease in survival, with only 17% cell viability. Notably, Srl alone at equivalent concentrations did not induce significant cytotoxicity, clearly indicating the synergistic effect of *ER*-Cu_1_SAZyme in combination with Srl. This synergistic effect was further validated by live/dead cell staining analysis. As shown in Figure 4C, co-staining with Calcein-AM (green fluorescence for live cells) and PI (red fluorescence for dead cells) revealed a substantial increase in dead cells in the *ER*-Cu_1_SAZyme-treated group, while the Srl/*ER*-Cu_1_SAZyme group displayed almost no green fluorescence, indicating near-complete cell death. Since dying cells lose their spread morphology and become more rounded, they may appear slightly smaller under identical magnification. These results demonstrate that Srl/*ER*-Cu_1_SAZyme significantly enhances antitumor efficacy, offering a promising strategy for therapeutic interventions.

To investigate whether the therapeutic effects of Srl/*ER*-Cu_1_SAZyme are associated with ROS-mediated cell damage, intracellular ROS levels were measured. Using the green fluorescent probe 2,7-dichlorofluorescein diacetate (DCFH-DA), a significant increase in ROS levels was observed following *ER*-Cu_1_SAZyme treatment. Specifically, ROS levels in Cal-27 cells increased from 5.59% to 61.78%, demonstrating that Srl substantially enhanced the catalytic activity of *ER*-Cu_1_SAZyme in tumor cells (Figure 4D). This increase in ROS was also confirmed by stronger fluorescence signals observed in confocal microscopy after treatment with *ER*-Cu_1_SAZyme and Srl/*ER*-Cu_1_SAZyme (Figure 4E). Additionally, the role of Srl/*ER*-Cu_1_SAZyme in boosting ROS generation was further explored by measuring intracellular GSH levels. While Srl alone had no significant effect on GSH levels, *ER*-Cu_1_SAZyme reduced GSH levels to approximately 40%, and in the Srl/*ER*-Cu_1_SAZyme group, GSH dropped further to about 27% (Figure 4F). This confirms that *ER*-Cu_1_SAZyme exhibits potent catalytic activity, effectively depleting GSH and significantly amplifying oxidative stress in cancer cells with the assistance of Srl. Furthermore, malondialdehyde (MDA), a key marker of oxidative stress, was measured. MDA levels in the *ER*-Cu_1_SAZyme group were 2.6 times higher than in the control group, and this increased further to 4.3 times in the Srl/*ER*-Cu_1_SAZyme group (Figure 4G). These results highlight the exceptional catalytic performance of Srl/*ER*-Cu_1_SAZyme in amplifying oxidative stress within cancer cells, thereby enhancing its potent therapeutic effect.

After evaluating ROS levels, the effect of Srl/*ER*-Cu_1_SAZyme on mitochondria was investigated. Mitochondria are not only the primary source of ROS in cells but also major targets of ROS-induced damage, which can lead to ferroptosis in cancer cells 39. Mitochondrial damage was assessed by measuring the mitochondrial membrane potential (MMP) using the JC-1 fluorescent probe. In healthy cells, JC-1 aggregates in the mitochondrial matrix, emitting red fluorescence. However, in damaged cells with depolarized mitochondrial membranes, JC-1 remains in its monomeric form, emitting green fluorescence 40. Following treatment with Srl/*ER*-Cu_1_SAZyme, a significant increase in green fluorescence and a reduction in red fluorescence were observed, indicating severe mitochondrial damage caused by ROS generated by the nanomedicine (Figures 4H and S20). These results suggest that Srl/*ER*-Cu_1_SAZyme effectively depletes GSH and generates ROS, inducing oxidative stress that results in mitochondrial dysfunction. Consequently, Srl/*ER*-Cu_1_SAZyme-induced cancer cell death appears to be linked to ROS-mediated oxidative damage and ferroptosis.

### Srl/*ER*-Cu_1_SAZyme induces robust ferroptosis in tumor cells

**Figure 5:**
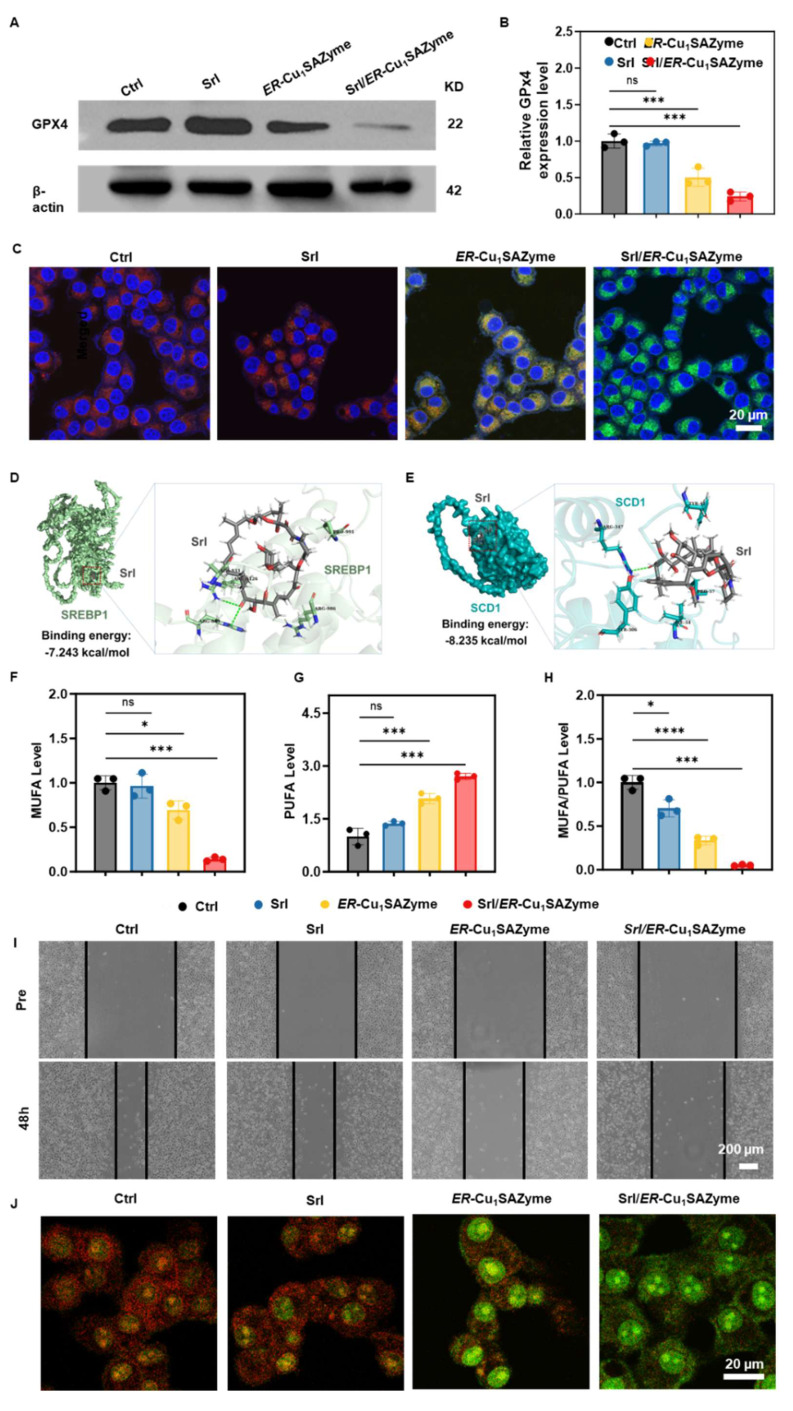
Potential antitumor mechanism of *ER*-Cu_1_SAZyme. (A) Western blot analysis of GPX4 expression in Cal-27 cells treated with Srl/*ER*-Cu_1_SAZyme. (B) Quantification of GPX4 protein levels normalized to untreated controls, with β-actin as a loading control. Band intensities were analyzed using ImageJ software and presented as fold change relative to control. (C) Lipid peroxidation levels measured by BODIPY-C11 staining in Cal-27 cells after Srl/*ER*-Cu_1_SAZyme treatment. Red fluorescence indicates non-oxidized lipids, while green fluorescence represents oxidized lipids. (D-E) Molecular docking schematic: Structural formulas of Srl, SREBP1 (D), and SCD1 (E), with close-up views of the interaction sites. (F-H) Intracellular levels of MUFA (F), PUFA (G), and the MUFA/PUFA ratio (H) in Cal-27 cells following Srl/*ER*-Cu_1_SAZyme treatment. (I) Representative images of Cal-27 cell migration at 48 hours after Srl/*ER*-Cu_1_SAZyme treatment. (J) CLSM images of Cal-27 cells stained with AO after Srl/*ER*-Cu_1_SAZyme treatment. Red fluorescence indicates intact lysosomes, while green fluorescence reflects damaged lysosomes. Data are presented as mean ± s.d. All measurements (n) are biologically independent.

Building on the observed catalytic performance of Srl/*ER*-Cu_1_SAZyme, its capacity to induce ROS-mediated ferroptosis was further assessed by examining key markers such as GPX4 expression and LPO levels. GPX4 plays a critical role in regulating cellular redox homeostasis and is directly associated with ferroptosis. Since GSH is essential for GPX4 synthesis, its depletion naturally leads to reduced GPX4 levels 41. As anticipated, *ER*-Cu_1_SAZyme significantly reduced GPX4 expression to 40%, and treatment with Srl/*ER*-Cu_1_SAZyme further decreased it to 20%, reflecting a substantial disruption of cellular redox balance induced by this nanoformulation (Figures 5A, B, and S21). LPO, a key hallmark of ferroptosis, was also assessed. Intracellular MDA levels, a terminal product of LPO, were significantly elevated in Srl/*ER*-Cu_1_SAZyme-treated cells, indicating heightened oxidative stress. This increase in MDA suggests considerable LPO in the cell membrane, consistent with ferroptotic cell death 42. To directly measure LPO, the BODIPY-C11 fluorophore was used. This probe undergoes oxidation, resulting in a fluorescence shift from red (reduced state) to green (oxidized state), making it widely applicable in ferroptosis and oxidative stress studies 43, 44. A distinct green fluorescence was observed in Srl/*ER*-Cu_1_SAZyme-treated cells, confirming high levels of LPO (Figures 5C and S22). These results validate that Srl/*ER*-Cu_1_SAZyme-induced cancer cell death is closely linked to ferroptosis, driven by oxidative stress and membrane LPO.

To further confirm the catalytic LPO activity of *ER*-Cu_1_SAZyme, a model liposome system was established. As shown in Figure S23, untreated liposomes and those treated with *ER*-Cu_1_SAZyme or H_2_O_2_ alone emitted only red fluorescence around 590 nm, corresponding to the reduced form of the liposome. In contrast, the *ER*-Cu_1_SAZyme plus H_2_O_2_ group displayed a marked increase in green fluorescence, indicating that *ER*-Cu_1_SAZyme effectively catalyzes LPO in the liposome model. This supports its role in promoting membrane lipid oxidation during ferroptosis.

To elucidate the underlying mechanism, the regulation of lipid metabolism by Srl/*ER*-Cu_1_SAZyme was assessed, as the cell membrane, a primary target of LPO, plays a pivotal role in determining sensitivity to LPO 13. SREBP1, a transcription factor involved in lipid metabolism, is regulated by mTOR and drives the expression of SCD1 45-47. SCD1, in turn, promotes the synthesis of MUFAs, which incorporate into the cell membrane, replacing PUFAs 48. This shift reduces LPO and enhances resistance to ferroptosis 49. To evaluate the effect of Srl on modulating membrane composition, molecular docking studies were conducted between Srl and key lipid metabolic proteins. As previously reported, Srl exhibits high affinity for mTOR 50, with a binding energy of -8.442 kcal/mol, indicating potent inhibition (Figure S24 and Tables S3-S4). Additionally, Srl showed strong binding affinity for SREBP1 and SCD1, with binding energies of -7.243 and -8.235 kcal/mol, respectively (Figures 5D, E and S25-26). The interacting residues between Srl and SREBP1 or SCD1 are listed in Tables S5-S6. These interactions suggest that Srl may interfere with MUFA content in the cell membrane. To further explore this, ELISA was used to measure MUFA levels in cells. The results demonstrated significant inhibition of MUFA synthesis in the Srl/*ER*-Cu_1_SAZyme group, highlighting the nanomedicine's effective therapeutic action (Figure 5F). In contrast, free Srl exhibited limited efficacy, likely due to its hydrophobic nature, which hinders bioavailability at low concentrations in physiological environments. Conversely, the Srl/*ER*-Cu_1_SAZyme nanomedicine enhanced cellular uptake, improving its therapeutic effectiveness. Furthermore, an unexpected increase in PUFA levels was observed after treatment with Srl/*ER*-Cu_1_SAZyme (Figure 5G). This could result from the reduction in MUFA and increased oxidative stress, which likely activates a compensatory mechanism to elevate PUFA levels, thereby helping cells mitigate the enhanced oxidative stress 51, 52. This observation highlights the remarkable plasticity and survival capacity of cancer cells.

High-performance liquid chromatography-tandem mass spectrometry (HPLC-MS/MS) was employed to further analyze the lipid composition of the cell membrane. Five major membrane-associated lipid classes were examined: phosphatidylcholine (PC), phosphatidylethanolamine (PE), phosphatidylserine (PS), cholesteryl ester (CE), and sphingomyelin (SM), all of which are essential for membrane integrity and regulate ferroptosis sensitivity 53-57. For each lipid class, the relative abundance of MUFA- and PUFA-containing species was quantified, and the MUFA/PUFA ratio was calculated. As shown in Figure S27, treatment with Srl/*ER*-Cu_1_SAZyme led to a reduction in MUFA/PUFA ratios across multiple lipid classes. Similar findings were confirmed by the ELISA assay (Figure 5H), indicating that Srl/*ER*-Cu_1_SAZyme modulates lipid metabolism, promotes LPO, and enhances ferroptosis.

Given the multifaceted nature of tumor cell death, the observed lipid-centered metabolic changes, along with significant LPO and multiple ferroptosis-associated molecular markers, strongly suggest that ferroptosis plays a critical role in the cellular response induced by Srl/*ER*-Cu_1_SAZyme. Apoptosis may also contribute, albeit to a lesser extent, as indicated by the partial rescue effect observed with the apoptosis inhibitor Z-VAD-FMK (Figure S28). In contrast, ferroptosis inhibition using Ferrostatin-1 provided a much stronger protective effect, further emphasizing ferroptosis as the dominant mechanism underlying the observed cytotoxicity.

Furthermore, scratch assays revealed that Srl/*ER*-Cu_1_SAZyme effectively inhibits cancer cell migration, suggesting that the compensatory increase in PUFA is insufficient to induce metastasis, thus enhancing therapeutic efficacy (Figures 5I and S29). Significant lysosomal damage was also observed. As shown in Figure 5J, Srl alone had minimal impact on lysosomal integrity, but under the strong oxidative stress induced by *ER*-Cu_1_SAZyme, a notable increase in lysosomal internal pH was observed, as evidenced by the diminished red fluorescence, indicating disruption of the lysosomal membrane. In the Srl/*ER*-Cu_1_SAZyme group, Srl's inhibition of mTOR-mediated lysosomal regeneration, combined with the potent oxidative damage from *ER*-Cu_1_SAZyme, exacerbated lysosomal dysfunction, as reflected by the complete loss of red fluorescence. Lysosomal damage impairs the degradation of cellular debris and damaged organelles, leading to the accumulation of toxic intermediates 58, 59. Therefore, lysosomal destruction disrupts the tumor cells' ability to repair and maintain cellular homeostasis, reducing drug resistance and increasing sensitivity to ferroptosis.

### The ferroptosis sensitization mechanism of the Srl/*ER*-Cu_1_SAZyme

**Figure 6:**
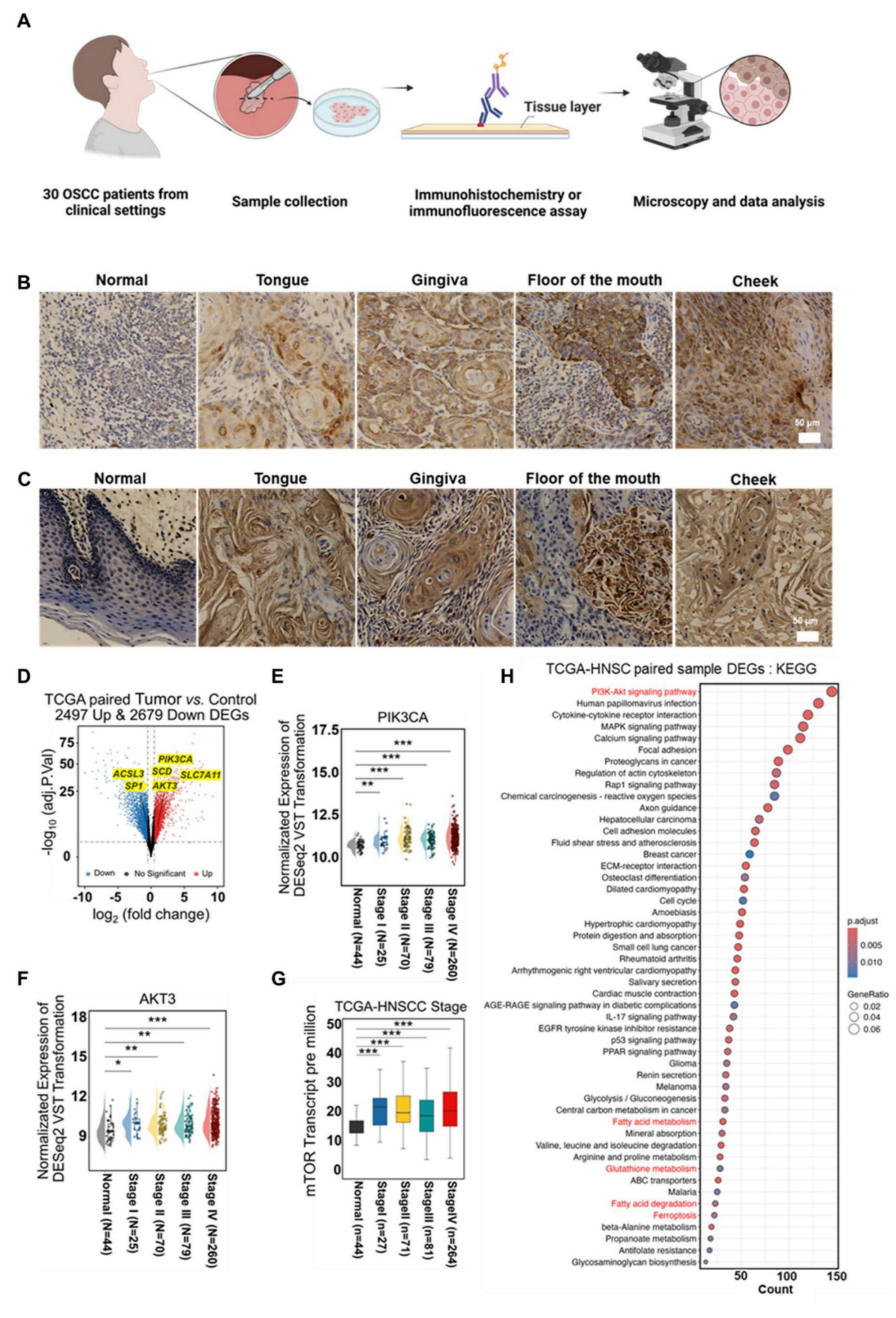
Abnormalities in SCD1 and its upstream regulators in clinical patient tissue samples. (A) Schematic of clinical cancer tissue collection and analysis. (B) Representative image of SCD1 expression in OSCC tissues from 30 paired clinical samples. (C) Representative image of mTOR expression in OSCC tissues from 30 paired clinical samples. (D) Volcano plot comparing gene expression between Tumor and Control groups, with blue indicating downregulated DEGs and red indicating upregulated DEGs. (E-G) Expression of PIK3CA (E), AKT3 (F), and mTOR (G) across individual cancer stages in HNSCC. (H) DEG enrichment in KEGG pathways. Data are presented as mean ± s.d. *P* < 0.001, ***, *P* < 0.01, **, *P* < 0.05, *.

After confirming that Srl/*ER*-Cu_1_SAZyme effectively induces ferroptosis in Cal-27 cells, the intrinsic mechanisms by which Srl/*ER*-Cu_1_SAZyme modulates lipid metabolism to enhance ferroptosis susceptibility were further investigated. To better simulate clinical conditions, 30 clinical samples from patients with OSCC were collected (Figure 6A and Table S7). Immunohistochemistry revealed marked overexpression of SCD1 in OSCC tissues from various anatomical sites, including the buccal mucosa, floor of mouth, gingiva, and tongue (Figures 6B and S30-31), alongside moderate increases in GPX4 (Figures S32-33). These results suggest that OSCC cells may resist ferroptosis by activating the MUFA synthesis pathway and enhancing antioxidant defense mechanisms, thus maintaining cell survival and proliferation. Specifically, the overexpression of SCD1 drives the conversion of saturated fatty acids to MUFAs, which reduces LPO mediated by PUFAs, while elevated GPX4 levels further inhibit ferroptosis by scavenging LPO. Moreover, significant overexpression of mTOR, an upstream regulator of SCD1, was observed (Figures 6C and S34-35), likely promoting SCD1 transcription *via* the activation of SREBP1 and thereby reinforcing a metabolic phenotype that resists ferroptosis.

To further validate this hypothesis and enhance the clinical relevance of the findings, a comprehensive analysis was conducted using data from the TCGA (The Cancer Genome Atlas) and CPTAC (Clinical Proteomic Tumor Analysis Consortium) databases. Gene expression differences between normal and tumor tissues were compared systematically. Since OSCC represents the sixth most common cancer globally, accounting for over 90% of head and neck malignancies 60, data from head and neck squamous cell carcinoma (HNSCC) were also included to increase sample size and ensure statistically significant results. Comparative transcriptomics of paired tumor and normal tissues from TCGA revealed 2,497 upregulated genes and 2,679 downregulated genes in tumor tissues (Figure 6D). Key alterations included lipid metabolism-related genes (ACSL3, SCD1), redox homeostasis modulators (SLC7A11), and oncogenic drivers (PIK3CA, AKT3, SP1), suggesting significant rewiring of lipid metabolism, activation of pro-invasive signaling, and disruption of oxidative stress balance. Further analysis revealed a significant upregulation of SCD1 upstream regulators, including PIK3CA, AKT3, and mTOR, across all stages of HNSCC (Figures 6E-G).

Notably, mTOR expression exhibited a progressive increase with age (Figure S36) and differed by sex (Figure S37), with particularly high levels observed in lymph node metastases (Figure S38). Validation using TCGA and CPTAC datasets confirmed significantly higher mTOR expression in primary tumors compared to normal tissues (Figures S39-40). These results suggest that sustained activation of SCD1 upstream genes may contribute to tumor progression by promoting cell proliferation and survival through lipid metabolic reprogramming. Subsequently, differentially expressed genes (DEGs) underwent Kyoto Encyclopedia of Genes and Genomes (KEGG) pathway analysis (Figure 6H), revealing enrichment in pathways related to PI3K-AKT signaling, fatty acid metabolism, GSH metabolism, fatty acid degradation, and ferroptosis. These results provide strong theoretical support for targeting lipid metabolism remodeling as a potential strategy to enhance ferroptosis and improve therapeutic outcomes.

The identification of mutations and altered expression levels of MUFA-related genes in clinical tissue samples of patients with OSCC further substantiates the potential of lipid metabolism modulation to improve therapeutic outcomes in OSCC. Previous cellular data have demonstrated that Srl/*ER*-Cu_1_SAZyme effectively reduces the MUFA/PUFA ratio and activates ferroptosis in Cal-27 cells. To investigate the underlying mechanisms and assess the clinical relevance, gene and protein expression levels in Cal-27 cells treated with Srl/*ER*-Cu_1_SAZyme were analyzed to identify the molecular factors driving this therapeutic effect.

**Figure 7:**
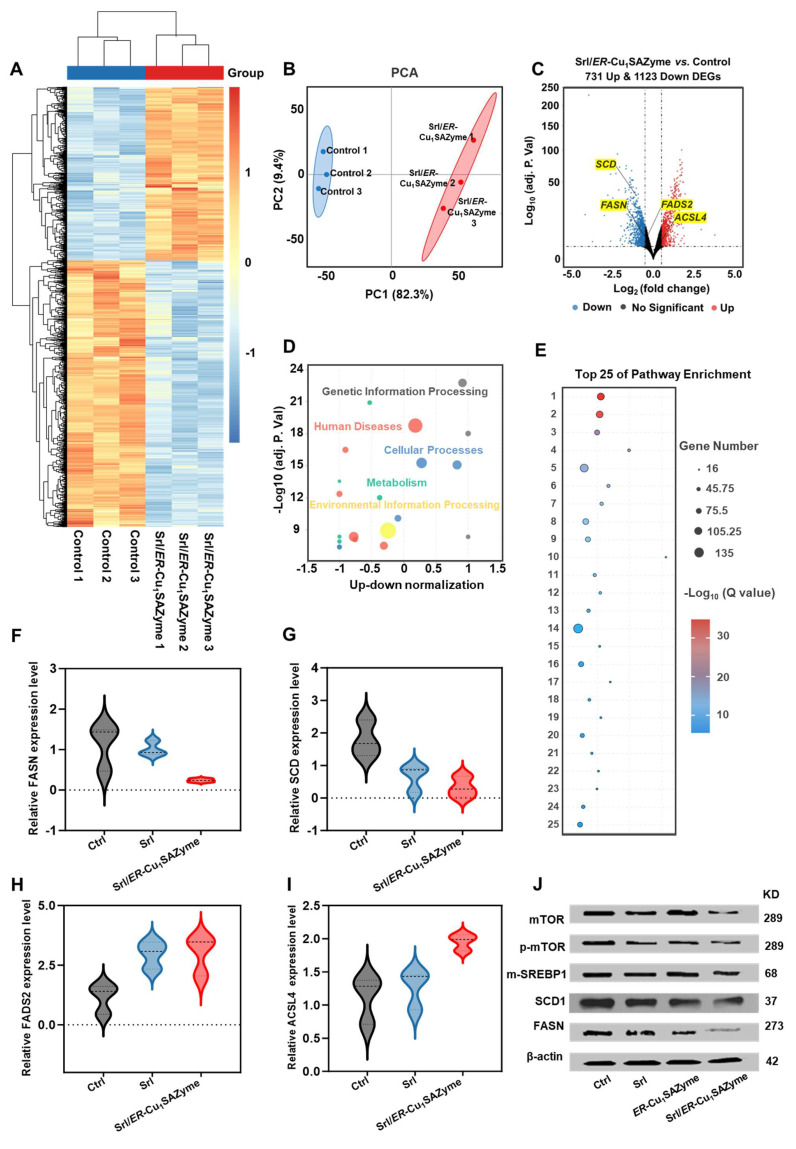
Gene and protein regulation of Cal-27 cells by *ER*-Cu_1_SAZyme. (A) Heat map showing intracellular transcriptomic regulation in Srl/*ER*-Cu_1_SAZyme-treated Cal-27 cells. Blue indicates downregulated DEGs, red indicates upregulated DEGs. (B) PCA of transcriptomics sequencing comparing the Control and Srl/*ER*-Cu_1_SAZyme groups. (C) Volcano plot comparing gene expression between Control and Srl/*ER*-Cu_1_SAZyme groups. Blue indicates downregulated DEGs, red indicates upregulated DEGs. (D) Pathway enrichment analysis of Srl/*ER*-Cu_1_SAZyme-treated Cal-27 cells. (E) Top 25 pathways enriched after Srl/*ER*-Cu_1_SAZyme treatment. (F-I) qPCR analysis of gene expression in Srl/*ER*-Cu_1_SAZyme-treated Cal-27 cells. (J) Western blot analysis of mTOR, p-mTOR, m-SREBP1, SCD1, and FASN expression in Srl/*ER*-Cu_1_SAZyme-treated Cal-27 cells. Data are presented as mean ± s.d., and all measurements (n) are biologically independent.

As shown in Figure 7A, the heatmap reveals significant differences in gene expression between the Srl/*ER*-Cu_1_SAZyme-treated and control groups. Principal component analysis (PCA) in Figure 7B confirms clear separation between the gene expression profiles of Srl/*ER*-Cu_1_SAZyme-treated and control cells, indicating that treatment significantly alters cellular gene expression. The heatmap (Figure S41) and volcano plot (Figure 7C) show 731 upregulated and 1123 downregulated DEGs in the Srl/*ER*-Cu_1_SAZyme-treated group compared to controls. Notably, downregulation of key genes such as FASN, SCD, and FADS2 was observed, all of which are involved in the SREBP1/SCD1-mediated synthesis of MUFAs. This downregulation leads to reduced MUFA levels. Conversely, ACSL4, which facilitates the incorporation of PUFAs into membrane phospholipids, was upregulated, suggesting an increased incorporation of PUFAs into the cell membrane. Combined with reduced MUFA synthesis, this shift lowers the MUFA/PUFA ratio in the membrane, increasing its susceptibility to LPO. These gene alterations impact several biological processes, including metabolism, human diseases, genetic information processing, and cellular functions (Figure 7D).

Pathway enrichment analysis further highlighted the involvement of processes related to cancer, cell cycle regulation, DNA repair, oxidative stress, and ferroptosis (Figure 7E), suggesting that Srl/*ER*-Cu_1_SAZyme-induced lipid metabolic reprogramming may influence these critical pathways. These findings were corroborated by qPCR analysis (Figures 7F-I, Table S8), which provided additional validation of gene expression changes, supporting the hypothesis that Srl/*ER*-Cu_1_SAZyme sensitizes cells to oxidative stress through lipid metabolism alterations. Western blot analysis (Figures 7J, S42, S43) further confirmed these changes at the protein level. Compared to the control group, the Srl/*ER*-Cu_1_SAZyme-treated group exhibited significantly reduced protein expression of SCD1 (Figure S44) and FASN (Figure S45), consistent with observed gene expression changes. Additionally, the expression of upstream regulators of SCD1, including mTOR (Figure S46) and SREBP1 (Figure S47), was notably decreased, providing further evidence that Srl/*ER*-Cu_1_SAZyme enhances ferroptosis sensitivity by modulating lipid metabolic pathways.

In conclusion, combined Srl/*ER*-Cu_1_SAZyme treatment enhances tumor cell sensitivity to ferroptosis by reprogramming lipid metabolism, particularly by modulating the overactive MUFA synthesis and reshaping the MUFA/PUFA ratio in the cell membrane. This effect is achieved through the suppression of SCD1-mediated MUFA synthesis *via* upstream regulators, such as SREBP1 and mTOR, while simultaneously promoting the incorporation of PUFAs into the membrane by upregulating ACSL4. This reprogramming of the cell membrane lipid composition increases ferroptosis sensitivity, enhancing LPO induced by the catalytic action of Srl/*ER*-Cu_1_SAZyme-generated •OH, thereby robustly inducing ferroptosis.

### *In vivo* antitumor efficacy of the Srl/*ER*-Cu_1_SAZyme

**Figure 8:**
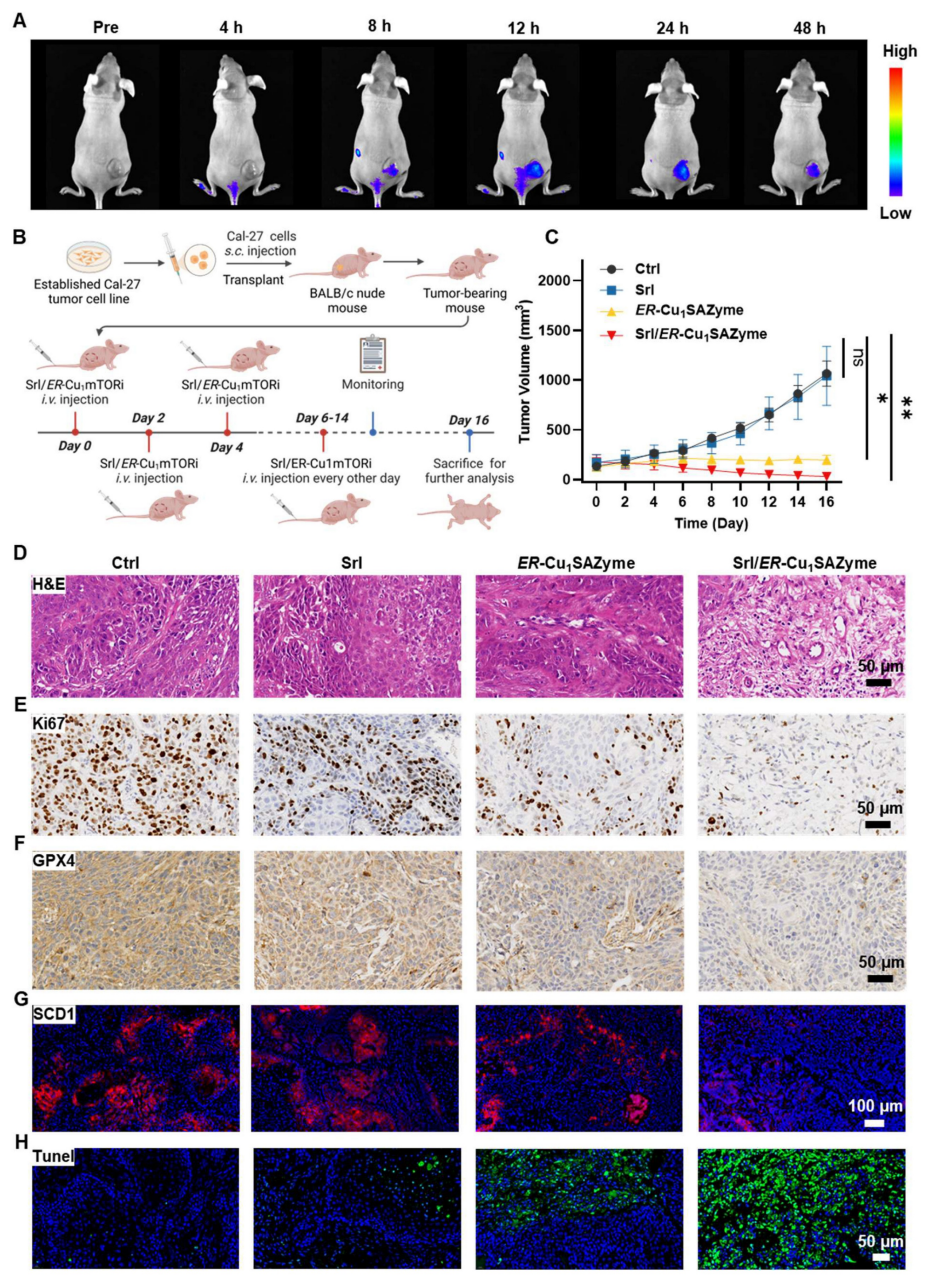
Efficient therapeutic efficacy against Cal-27 tumors by *ER*-Cu_1_SAZyme. (A) Real-time biodistribution images of fluorescently labeled Srl/*ER*-Cu_1_SAZyme before and after injection. (B) Illustration of treatment schedule. This Figure was created on Biorender.com. (C) Tumor growth curves of individual mice in each group (n = 5 biologically independent mice per group). (D-H) Histological microscopic images of dissected tumors stained with H&E (D), Ki67 (E), GPX4 (F), SCD1 (G), and TUNEL (H). Data are presented as mean ± s.d., and all measurements (n) are biologically independent.

Following the elucidation of the cancer cell-killing mechanism, the *in vivo* antitumor efficacy of Srl/*ER*-Cu_1_SAZyme was evaluated using a Balb/c nude mouse model bearing Cal-27 tumors. To establish a foundation for assessing the antitumor activity, the biodistribution of the nanotherapeutic agent was first investigated. As shown in Figure 8A, fluorescently labeled *ER*-Cu_1_SAZyme began to accumulate in tumor tissues 8 hours post-injection, peaking around 24 hours. Notably, significant amounts of the nanomedicine persisted in the tumor tissue even after 48 hours, indicating prolonged retention that could support sustained therapeutic action. To further confirm the *in vivo* distribution, ex *vivo* biodistribution analysis was conducted 48 hours after administration (Figure S48). The fluorescence signal was primarily detected in tumor tissues, with only low-level accumulation in the liver and negligible signals in other major organs, including the heart, spleen, lungs, and kidneys. This distribution pattern aligns with the anticipated clearance behavior of systemically administered nanomaterials and highlights the favorable tumor selectivity of Srl/*ER*-Cu_1_SAZyme 61, 62.

After confirming the accumulation of Srl/*ER*-Cu_1_SAZyme in tumors, its antitumor activity was rigorously assessed. Mice were randomly divided into four groups: Saline, Srl, *ER*-Cu_1_SAZyme, and Srl/*ER*-Cu_1_SAZyme. Treatments were administered *via* tail vein injections every other day for a total of eight doses to evaluate efficacy (Figure 8B). Tumor growth curves revealed that Srl alone had a negligible effect on tumor progression, while *ER*-Cu_1_SAZyme inhibited growth through catalytic damage, though it did not fully halt tumor development. In stark contrast, the combination treatment of Srl/*ER*-Cu_1_SAZyme resulted in significant tumor growth suppression, with tumors in this group being approximately 33 times smaller than those in the Saline group and 3.7 times smaller than their initial size (Figures 8C and S49-51). These results suggest that Srl enhances the catalytic therapeutic effect of *ER*-Cu_1_SAZyme, highlighting the combination treatment as a promising antitumor strategy.

The enhanced antitumor efficacy of Srl/*ER*-Cu_1_SAZyme was further validated through histological analysis. Tumors treated with Srl/*ER*-Cu_1_SAZyme exhibited more extensive necrosis and cavitation compared to those treated with *ER*-Cu_1_SAZyme alone, consistent with the tumor growth data (Figure 8D). The Ki67 proliferation marker, indicative of tumor cell proliferation, was significantly reduced in the Srl/*ER*-Cu_1_SAZyme-treated group, signifying widespread tumor cell death and suppressed proliferation (Figure 8E). To assess the role of ferroptosis, GPX4 expression levels were analyzed (Figure 8F). Both *ER*-Cu_1_SAZyme and Srl/*ER*-Cu_1_SAZyme treatments downregulated SCD1 expression, with the latter exhibiting a more pronounced effect, further supporting the cellular-level findings. This suggests that the inhibition of SCD1 disrupts MUFA synthesis, thereby sensitizing cells to ferroptosis induced by ROS damage mediated by *ER*-Cu_1_SAZyme, demonstrating the therapeutic efficacy *in vivo*. Notably, Srl/*ER*-Cu_1_SAZyme was more effective at reducing GPX4 levels compared to *ER*-Cu_1_SAZyme, further confirming the superior therapeutic potential of this ferroptosis-based approach for treating OSCC (Figures 8G and S52).

Additionally, apoptosis also contributed to the therapeutic effect, as confirmed by TUNEL staining. A significant increase in green fluorescent cells was observed in the *ER*-Cu_1_SAZyme-treated group, with an even higher number in the Srl/*ER*-Cu_1_SAZyme group (Figure 8H). Similar results were observed with Caspase-3 staining, where a significant number of positive cells were detected in the Srl/*ER*-Cu_1_SAZyme-treated tumors (Figure S53). These results suggest that the disruption of tumor cell metabolism reduces their adaptability and promotes apoptosis 63. These results indicate that the Srl/*ER*-Cu_1_SAZyme strategy induces a complex and multifaceted tumor cell death process, making it a promising and potent approach for cancer treatment.

### The biosafety of the Srl/*ER*-Cu_1_SAZyme

**Figure 9:**
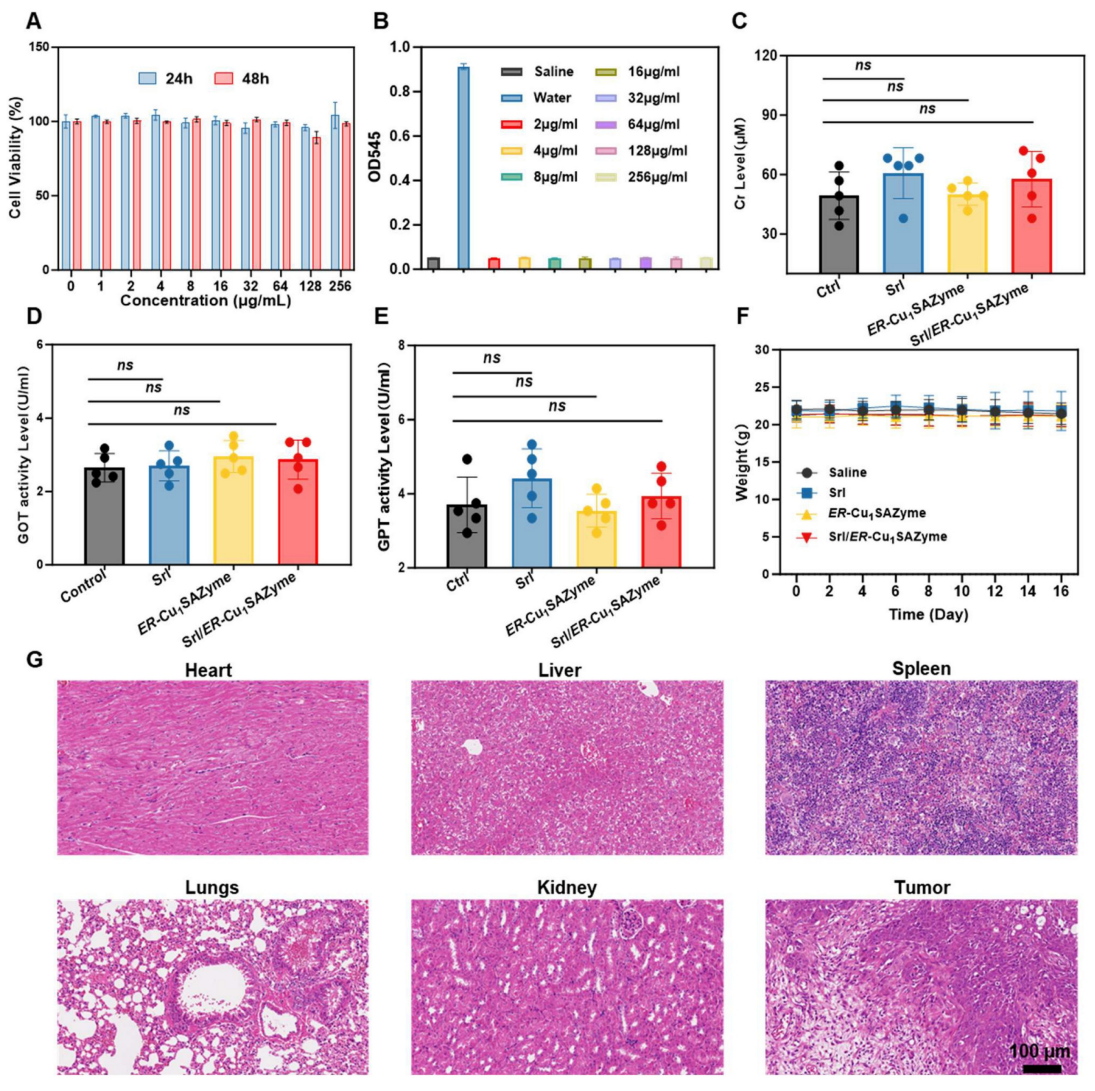
*In vivo* safety of *ER*-Cu_1_SAZyme. (A) Cell viability of HK-2 cells treated with Srl/*ER*-Cu_1_SAZyme for 24 and 48 hours. (B) Hemolysis test of Srl/*ER*-Cu_1_SAZyme at different concentrations. (C-E) Serum biochemistry parameters, including Cr (C), GOT (D), and GPT (E) levels, measured on the 16th day post-administration. (F) Body weight changes in mice after treatment. (G) Histological microscopic images of tissues stained with H&E. Data are presented as mean ± s.d., and all measurements (n) are biologically independent.

The biosafety of Srl/*ER*-Cu_1_SAZyme is a key factor for its potential application in nanodrug therapies, necessitating a thorough evaluation of its biocompatibility and safety profile. To assess potential kidney damage, human proximal tubule epithelial (HK-2) cells were co-incubated with Srl/*ER*-Cu_1_SAZyme. Results indicated that the viability of HK-2 cells remained largely unaffected, even at high concentrations of 256 μg/ml over a 48-hour exposure period, demonstrating the excellent biosafety of Srl/*ER*-Cu_1_SAZyme for normal cells (Figure 9A). To further assess hemocompatibility, hemolysis assays were conducted, as red blood cells are among the first to encounter nanodrugs in circulation (Figure 9B). The results showed no hemolysis at concentrations up to 256 μg/ml, indicating minimal acute hepatotoxicity associated with this nanotherapeutic agent.

For *in vivo* hepatorenal safety evaluation, serum samples were collected at the end of treatment, and levels of creatinine, along with liver function markers such as glutamic-pyruvic transaminase (GPT) and glutamic oxaloacetic transaminase (GOT), were measured (Figures 9C-E). All parameters remained within the normal range, suggesting minimal liver and kidney toxicity associated with Srl/*ER*-Cu_1_SAZyme administration. Moreover, the steady weight gain observed in treated mice further supported the well-tolerated nature of the treatment (Figure 9F).

Histological analysis of various organs (heart, liver, spleen, lung, and kidney) was performed *via* H&E staining post-treatment. No significant tissue damage was detected, supporting the favorable *in vivo* safety profile and efficient clearance behavior of the treatment (Figure 9G). These results strongly suggest that Srl/*ER*-Cu_1_SAZyme exhibits excellent biocompatibility and biosafety *in vivo*.

## Conclusions

In this study, a novel edge-rich Cu-N_3_ single-atom nanozyme (*ER*-Cu_1_SAZyme) was developed, characterized by its unique hollow, porous structure and catalytic centers enriched at the edges. This distinct architecture enhances catalytic activity, enabling efficient ROS generation that disrupts cellular redox homeostasis. In combination with the FDA-approved drug Srl, *ER*-Cu_1_SAZyme synergistically modulates lipid metabolism in tumor cells by inhibiting SCD1-mediated MUFA synthesis and promoting ACSL4-mediated PUFA activation. This lipid remodeling alters the tumor cell membrane composition, enhancing susceptibility to LPO and ferroptosis. In tumor-bearing murine models, treatment with Srl/*ER*-Cu_1_SAZyme resulted in a significant reduction in tumor volume, demonstrating its potent therapeutic efficacy with minimal toxicity. These results highlight the potential of targeting lipid metabolic reprogramming combined with enhanced ROS generation as an effective strategy to overcome tumor resistance and improve ferroptosis-based therapies. This study offers new insights and a strong data foundation for the design and application of novel single-atom nanozymes, paving the way for their future clinical translation in biomedical applications.

## Supplementary Material

Supplementary methods, figures and tables.

## Data Availability

The corresponding author can provide the data supporting this study's findings upon a reasonable request.

## Abbreviations

ACSL4: acyl-CoA synthetase long-chain family member 4
BET: Brunauer-Emmett-Teller
CPTAC: Clinical Proteomic Tumor Analysis Consortium
DEGs: differentially expressed genes
DFT: density functional theory
DOS: density of states
*ER*-Cu_1_SAZyme: edge-rich Cu-N_3_ single-atom nanozyme
ESR: electron spin resonance
EXAFS: extended X-ray absorption fine structure
GSH: glutathione
HAADF-STEM: high-angle annular dark-field scanning transmission electron microscopy
HNSCC: head and neck squamous cell carcinoma
H_2_O_2_: hydrogen peroxide
ICP-MS: inductively coupled plasma mass spectrometry
KEGG: Kyoto Encyclopedia of Genes and Genomes
LPO: lipid peroxidation
MDA: malondialdehyde
MMP: mitochondrial membrane potential
MUFAs: monounsaturated fatty acids
OSCC: oral squamous cell carcinoma
PCA: principal component analysis
PUFAs: polyunsaturated fatty acids
ROS: reactive oxygen species
SAZymes: single-atom nanozymes
SCD1: stearoyl-CoA desaturase 1
TEM: transmission electron microscopy
TCGA: The Cancer Genome Atlas
TME: tumor microenvironment
XANES: X-ray absorption near-edge structure
XAS: X-ray absorption spectroscopy
XPS: X-ray photoelectron spectroscopy
