# Supplementary material for: Edge-rich Cu-N3 single atom nanozyme drives lipid switching to potentiate tumor catalytic therapy
Source: Theranostics. 2026 Apr 8;16(11):6011–31. doi: 10.7150/thno.128026 (PMC13142117; doi:10.7150/thno.128026)
Supplement: Supplementary file 1 — Supplementary methods, figures and tables. [file thnov16p6011s1.pdf]

Supporting Information

Edge-rich Cu-N_3 single atom nanozyme drives lipid switching to potentiate tumor catalytic therapy

Xin Xing,[1]‡ Jiaji Yu,[2]‡ Yajie Zhang,[3]‡ Xue Chen,[1] Liang Chen,[1] Meng Du,[4] Jie Ding,[5] Zhiyi Chen,[4]* Li Li,[5]‡* Junjie Cheng[2]*

[a] Department of Stomatology, The First Affiliated Hospital of Wannan Medical College, Yijishan Hospital, Wuhu 241001, China

[b] Department of Nutrition and Food Hygiene, School of Public Health, Southeast University, Nanjing, 210009, China; E-mail: jjcheng@ustc.edu.cn; jjcheng@seu.edu.cn

[c] Central Laboratory, Department of Biobank, Nanjing Hospital of Chinese Medicine Affiliated to Nanjing University of Chinese Medicine, Nanjing 210022, China

[d] Key Laboratory of Medical Imaging Precision Theranostics and Radiation Protection, College of Hunan Province, Hengyang Medical School, The Affiliated Changsha Central Hospital, University of South China, Changsha, China. E-mail: zhiyi_chen@usc.edu.cn

[e] Max Planck Institute of Microstructure Physics, Weinberg 2, Halle, 06120, Germany
E-mail: lili@mpi-halle.mpg.de

†These authors contributed equally to this work.

Methods

Synthesis of *ER*-Cu_1SAZyme. To prepare *ER*-Cu_1SAZyme, mesoporous silica (SiO_2) was first synthesized using the sol-gel method as a template. SiO_2 nanoparticles were then mixed with dopamine (DA) and a copper precursor to initiate polymerization, forming a non-uniform polydopamine (PDA) coating around the SiO_2. After high-temperature pyrolysis at 800°C in an argon atmosphere for 4 h, the PDA coating converted into a nitrogen-doped carbon (CN) framework. Sodium hydroxide (NaOH, 2M) was subsequently used to etch the SiO_2 core, leaving behind a hollow structure within the PDA. 10 mg of *ER*-Cu_1SAZyme was sonicated in chloroform and 5 mg of DSPE-PEG_{5k} was slowly added to the solution at room temperature. The chloroform was then removed by rotary evaporation, the resulting film was rehydrated by aqueous solution and the resulting *ER*-Cu_1SAZyme was collected by centrifugation and washed three times with water. Specifically, the non-edge Cu_1SAZyme was prepared using the same precursor composition and processing steps as ER-Cu_1SAZyme, with the sole difference being a reduced pyrolysis temperature (700 °C instead of 800 °C). The similar procedure was used to prepare Srl/*ER*-Cu_1SAZyme and RF/*ER*-Cu_1SAZyme by mixing 1 mg Srl or IR780 in the chloroform.

Characterization of materials. The TEM images were collected using a JEM-2100F transmission electron microscope. The high-angle annular dark-field scanning transmission electron microscopy (HAADF-STEM) and corresponding eds elemental mapping images were performed on a FEI SPECTRA 300 with a dual-spherical aberration corrector, operated at 300 kV. X-ray photoelectron spectroscopy (XPS) was collected on scanning X-ray microprobe (PHI 5000, ULAC-PHI) using Al Ka radiation and the C1s peak at 284.8 eV as internal standard. XANES and EXAFS measurements were performed in transmission mode at the Japan Synchrotron Light Source (Spring-8) XAFCA beamline. Acquired XAFS data were processed with the ATHENA program and analyzed in the ARTEMIS program integrated with IFEFFIT software package. The size distribution of nanomaterials was measured *via* NanoBrook 90Plus PALS particle size analyzer.

Measurement of the POD-like activity of *ER*-Cu_1SAZyme, PEG-*ER*-Cu_1SAZyme and Cu_1SAZyme. To assess the POD-like activities of *ER*-Cu_1SAZyme, PEG-*ER*-Cu_1SAZyme and Cu_1SAZyme, a TMB assay was conducted. Different concentrations of *ER*-Cu_1SAZyme (0, 20, 40, 60, and 80 μg/mL) and 1.0 mM H_2O_2 were added to 0.05 M HAc-NaAc buffer (pH 6.0) followed by the addition of 1 mM TMB. After incubating the mixture for 5 minutes at 37°C, the absorbance was measured at 652 nm (8453, Agilent). Similarly, the effect of TMB/H_2O_2 solution concentrations on the catalytic reaction of *ER*-Cu_1SAZyme was determined by incubating 40 μg/mL *ER*-Cu_1SAZyme with varied TMB or H_2O_2 concentrations (0, 0.2, 0.4, 0.6, 0.8, and 1.0 mM). Additionally, the pH effect was assessed using HAc-NaAc buffer for pH 4.5 and 6.0, and PBS buffer for

pH 7.4. To measure the depletion of H_2O_2, a $Ti(SO_4)_2$ assay was utilized. Specifically, 2 mM H_2O_2 was incubated with 200 μg/mL Cu1/SAZyme in HAC-NaAC buffer (pH 6.0) at 37°C. After different incubation times, the mixtures were centrifuged, and 50 μL of the supernatant was mixed with 100 μL of $Ti(SO_4)_2$ working solution. The absorbance of the resulting solution was measured at 425 nm.

Kinetic measurements for the POD-like activity of *ER*-Cu_1SAZyme. To determine the kinetic parameters (Km and Vmax) of *ER*-Cu_1SAZyme, steady-state measurements were performed using a TMB-based colorimetric assay. The reaction was conducted in a pH 6.0 acetate buffer system, with a final concentration of 25 μg/mL *ER*-Cu_1SAZyme and 1 mM TMB. Hydrogen peroxide (H_2O_2) was used as the substrate, with a concentration gradient of 0.25, 0.5, 1, 4, and 8 mM. Reactions were initiated by adding H_2O_2 and monitored using a microplate reader. Absorbance at 652 nm was recorded over the first 5 minutes, and the initial reaction rates were calculated from the linear portion of the time–absorbance curves. The resulting data were fitted to the Michaelis-Menten and Lineweaver-Burk models to extract kinetic parameters.

Measurement of the CAT-, SOD-, or GPX-like activities of *ER*-Cu_1SAZyme. Catalase-like activity of *ER*-Cu_1SAZyme was evaluated using a dissolved oxygen meter. The reaction system contained 100 μg/mL *ER*-Cu_1SAZyme and 100 mM hydrogen peroxide (H_2O_2) in a suitable buffer. Upon addition of H_2O_2, the decomposition of H_2O_2 was monitored by measuring the increase in dissolved oxygen concentration over time. GPx-like activity was assessed using a commercial Total Glutathione Peroxidase Assay Kit (Beyotime, S0059S), based on the DTNB method. The assay was performed according to the manufacturer's instructions. Briefly, *ER*-Cu_1SAZyme was incubated with the reaction mixture containing glutathione and Cum-OOH substrate, and absorbance at 412 nm was recorded to quantify GPx-like activity. SOD-like activity was measured using the photochemical reduction of nitroblue tetrazolium (NBT). The reaction system (1.5 mL) consisted of 0.326 mM methionine, 0.15 mM EDTA-Na_2, 0.1125 mM NBT, and 0.05 mM riboflavin. *ER*-Cu_1SAZyme was added to achieve a final concentration of 40 μg/mL. The mixture was exposed to 4000 lux light at 25 °C for 20 minutes. A parallel reaction tube kept in the dark was used to zero the absorbance baseline. Absorbance at 560 nm was measured, and SOD-like activity was calculated based on the percentage inhibition of NBT photoreduction.

Lipid peroxidation (LPO) activity of *ER*-Cu_1SAZyme. The liposomes were prepared by the thin-film hydration method using lecithin as the phospholipid component, with BODIPY 581/591 C11 incorporated as a fluorescent reporter. Liposomes were then incubated with 100 μg/mL *ER*-Cu_1SAZyme and 0.5mM H_2O_2, and fluorescence changes were examined using confocal microscopy.

Targeted lipidomic analysis using HPLC-MS/MS. Spread the cells onto culture bottles and divide them into two groups: the control group and the drug treatment group. Then, treat the cells according to the previous treatment plan. After 24 h, collect 4×10^7 cells and collect cell membranes using a plasma membrane separation kit (Invent, China). The collected cell membranes were stored at -80 °C.Afterwards, the samples were sent to Tsingke Biotechnology for quantitative lipid sequencing.Lipids contents were detected by (http://www.metware.cn/) based on the AB Sciex QTRAP 6500 LC-MS/MS platform. After the detection is completed， unsupervised PCA (principal component analysis) was performed by statistics function prcomp within R (www.r-project.org)The data was unit variance scaled before unsupervised PCA. The HCA (hierarchical cluster analysis) results of samples and metabolites were presented as heatmaps with dendrograms, while pearson correlation coefficients (PCC) between samples were caculated by the cor function in R and presented as only heatmaps. Both HCA and PCC were carried out by R package pheatmap. For HCA, normalized signal intensities of metabolites (unit variance scaling) are visualized as a color spectrum. Differential metabolites were determined by VIP (VIP > 1) and P-value (Pvalue< 0.05, Student's t test). VIP values were extracted from OPLS-DA result, which also contain score plots and permutation plots, was generated using R package MetaboAnalystR. The data was log transform (log2) and mean centering before OPLS-DA. In order to avoid overfitting, a permutation test (200 permutations) was performed.

•OH detection. To perform the ESR experiment, 200 μg/mL *ER*-Cu_1SAZyme was added to a buffer solution (pH 6.0) containing 10 mM H_2O_2 and 100 μM DMPO. Superoxide dismutase was added to remove $O_2^{\bullet-}$ from the reaction. The mixture was transferred to a quartz tube and analyzed by ESR (JES-FA200, JEOL).

DFT computational details. The structural optimizations and energy calculations were performed within the framework of density functional theory (DFT). Generalized gradient approximation (GGA) with Perdew-Burke-Ernzerhof (PBE) method. Structural optimization and energy calculations were carried out to evaluate the catalytic models. The adsorption energies (E) were calculated according to the equation: $E = E_{tot} - (E_{sub} + E_{ad})$, where E_{tot} represents the total energy of the optimized system, the E_{sub} and E_{ad} represent the total energy of the clean substrate, and the total energy of the free adsorbate, respectively. The free energy was calculated using the equation: $\Delta G=\Delta E + \Delta ZPE - T\Delta S$, where ΔG represents free energy, ΔE represents total energy, ΔZPE represents zero-point energy and ΔTS represents entropic contributions.

GSH depletion. To measure GSH levels, DTNB was utilized. In HAC-NaAC buffer, 0.15 mM GSH was

incubated with 200 μg/mL *ER*-Cu_1SAZyme for different time periods. The mixtures were centrifuged, and the resulting supernatant (200 μL) was mixed with 300 μL of 2.5 mM DTNB working solution. The absorbance at 420 nm was measured using a spectrophotometer (8453, Agilent).

Collection and analysis of clinical samples. Tumor tissue sections and paired adjacent non-tumor tissue sections were collected from 30 patients with oral squamous cell carcinoma who underwent surgery in the Department of Oral and Maxillofacial Surgery, The First Affiliated Hospital of Wannan Medical College, between January 2022 and December 2024. The inclusion criteria were as follows: (1) initial radical surgery; (2) no history of other malignant tumors; and (3) no history of hematological diseases. The exclusion criteria were as follows: (1) palliative surgery; (2) missing covariate information; and (3) autoimmune disorders. The expression levels of SCD1, GPX4, and mTOR in tumor and paired adjacent non-tumor tissues were analyzed by immunohistochemistry and independently evaluated by two pathologists. Semi-quantitative evaluation was performed using the H-score method. Staining intensity was classified into four levels: 0, no staining; 1, weak staining; 2, moderate staining; and 3, strong staining. The H-score was calculated as follows: H-score = (% of cells with intensity 1 × 1) + (% of cells with intensity 2 × 2) + (% of cells with intensity 3 × 3). Expression was defined as negative (score < 10), weakly positive (10 ≤ score < 30), moderately positive (30 ≤ score < 70), or strongly positive (score ≥ 70). This study was approved by the Ethics Committee of The First Affiliated Hospital of Wannan Medical College (No. 2025KY-25) and was conducted in accordance with the Declaration of Helsinki. As this was a retrospective study, the requirement for informed consent was waived.

Cell culture. The oral squamous cell carcinoma (OSCC) cell line Cal-27 was sourced from Cellcook Biotech (Guangzhou, China). These cells were cultured in Dulbecco's Modified Eagle Medium (DMEM, Gibco, USA) supplemented with 10% fetal bovine serum (FBS, Corning, USA). The culture was maintained under a constant temperature and humidity in an incubator with a 5% CO_2 atmosphere at 37 °C. The utilized cell line is accompanied by an STR identification report, ensuring its authenticity.

Cellular cytotoxicity assays. The *via*bility of Cal-27 cells was assessed using a CCK-8 cell proliferation assay kit (Dojindo, Japan). Cells were harvested and plated in 96-well plates at a density of 5000 cells per well. Following an initial 12-hour incubation, various concentrations of *ER*-Cu_1SAZyme (0-80 μg mL^{-1}), Srl/*ER*-Cu_1SAZyme (0-80 μg mL^{-1}) or sirolimus (0-5.6 μg mL^{-1}) were introduced into fresh medium. The sirolimus concentration was standardized to correspond with the amount encapsulated within the Srl/*ER*-Cu_1SAZyme complex. After a 48-h incubation period, the medium was removed, and the wells were rinsed thrice PBS, Subsequently, 10 μL of CCK-8 reagent and 90 μL of fresh medium were added, followed by a 2-hour

incubation at 37 °C. The absorbance at 450 nm was measured using a microplate reader. Cell survival rates were calculated using the formula: (average optical density (OD) of treatment group / average OD of control group) × 100%.

Live/dead cell double staining assay. The presence of live and dead cells was determined using a Calcein/PI Live/ Dead *Via*bility/Cytotoxicity Assay Kit (Beyotime, China). Briefly, cells were grown in 6-well plates and treated with *ER*-Cu_1SAZyme (80 μg mL^{-1}), Srl/*ER*-Cu_1SAZyme (80 μg mL^{-1}) or sirolimus (5.6 μg mL^{-1}) for 48 h. Post- incubation, the medium was decanted, and an adequate volume of calcein AM/PI working solution was applied. The cells were then incubated in the dark at 37 °C for 30 minutes. The staining effect was examined under a fluorescence microscope (Calcein AM exhibits green fluorescence, PI exhibits red fluorescence).

Intracellular ROS detection. Intracellular ROS levels were measured using a reactive oxygen species assay kit (Jiancheng, China). Cells were plated at a density of 2.0×10^5 cells per well in 6-well plates and treated with *ER*-Cu_1SAZyme (40 μg mL-1), Srl/*ER*-Cu_1SAZyme (40 μg mL^{-1}) or sirolimus (2.8 μg mL^{-1}) for 24 h. Post-treatment, cells were loaded with the DCFH-DA fluorescent probe and incubated at 37 °C for 30 minutes. After collection and washing with PBS, the cells were analyzed using flow cytometry.

Detection of mitochondrial membrane potential. The mitochondrial membrane potential was assessed using an Enhanced Mitochondrial Membrane Potential Assay Kit with JC-1. Cells were treated as per the "Intracellular ROS detection" protocol and then incubated with JC-1 working solution for 20 minutes. After washing with PBS, the staining was observed under a laser confocal microscope. (JC-1 monomers exhibits green fluorescence, JC-1 aggregates exhibit red fluorescence).

Acridine orange staining. Cells were plated at a density of 2.0×10^5 cells per well in 6-well plates and treated with *ER*-Cu_1SAZyme (40 μg mL^{-1}), Srl/*ER*-Cu_1SAZyme (40 μg mL^{-1}), or sirolimus (2.8 μg mL^{-1}) for 24 h. After treatment, the cells were washed with PBS and incubated with acridine orange working solution for 30 min at room temperature in the dark. After washing with PBS, the cells were observed under a laser confocal microscope.

Detection of unsaturated fatty acid levels. The levels of monounsaturated fatty acids and polyunsaturated fatty acids in cells were measured using an ELISA method with kits from J&L Bio, China. Cell homogenates were prepared following the same protocol as in "Detection of lipid peroxidation level", and ELISA was performed according to the manufacturer's instructions.

Molecular docking. MTOR protein used 8RCK crystal structure in RCSB protein database, GPX4 protein used 6HN3 crystal structure in RCSB protein database, SCD1 protein used the structure with ID O00767 in UniProt database and the full-length structure predicted by AlphaFold; SREBP1 protein uses the structure with ID P36956 in UniProt database and the full-length structure predicted by AlphaFold. The above receptor molecules use pymol tool to remove crystal water, original ligand molecules, other free ions and heteroatoms from the molecules, and use ADFRsuite-1.0 to process, save the original charge of the protein, and generate pdbqt files for subsequent molecular docking. Rapamycin ligand molecular structure through the 2D structure provided in Aladdin's official website, using openbabel toolkit, using the steepest descent method, 1500 iterations, using MMFF94 force field to generate the 3D structure of small molecules, and using the Discovery Studio Visualizer v24.1.0.23298 tool to further optimize the 3D structure of the generated small molecules for subsequent molecular docking. The ligand structure was treated with ADFRsuite-1.0, the original charge of the molecule was preserved, and the pdbqt file was generated for docking. Molecular docking adopts global docking through AutoDock Vina software package, the center coordinates of the docking region for the MTOR protein are set to (229.366, 185.492, 217.195), with a grid point count of 159.803 × 100.232 × 146.05 in each XYZ direction; the center coordinates of the docking region for the GPX4 protein are set to (0.573, 0.552, 0.466), with a grid point count of 49.667×48.026×54.365 in each XYZ direction; the center coordinates of the docking region for the SCD1 protein are set to (6.975, 0.239, -5.716), with a grid point count of 88.42 × 69.731 × 74.424 in each XYZ direction; the center coordinates of the docking region for the SREBP1 protein are set to (-5.117, 20.625, -7.077), with a grid point count of 168.674 × 151.2 × 157.966 in each XYZ direction; the number of docking processes is set to 32, the grid step size is 0.375, and the other parameters adopt default values.

RNA extraction and quantitative PCR. The total RNA Extraction Kit (Tiangen, China) was used to extract the total RNA from the cells, and the cell treatment method was the same as the above. Then cDNA was synthesized by reverse transcription kit (Tiangen, China). Finally, the detection was carried out according to the instructions of fluorescent quantitative PCR kit (Biosharp, China). The obtained data were analyzed by relative quantitative method (Livak method). See Table S8 for primer sequence.

Mitochondrion, lysosome damage and lipid peroxide assay. Mitochondrial membrane potential, lysosomal damage, and lipid peroxidation assays. Cal-27 cells were seeded in confocal dishes for 24 h and then treated with Srl/*ER*-Cu_1SAZyme for the indicated time. After treatment, the cells were washed with PBS. Mitochondrial membrane potential was assessed using a JC-1 assay kit and confocal microscopy (LSM880,

Zeiss). Lysosomal damage was evaluated by acridine orange staining and fluorescence microscopy (IX71, Olympus). Lipid peroxidation was assessed using BODIPY 581/591 C11 staining followed by confocal microscopy (LSM880, Zeiss).

Intracellular GSH, and MDA levels. Cells were seeded in six-well culture plates and incubated for 24 h. Different formulations were then added and incubated for 24 h. Afterward, the cells were washed with PBS and analyzed for intracellular GSH, and MDA levels using the GSH and GSSG Assay Kit, and MDA Assay Kit (Beyotime), respectively.

Transcriptomics sequencing.

Heatmap scaling and centering data were generated by R package Scale (4.3.1) and performed by R package ComplexHeatmap (2.15.4). LogFC (log2 fold change) of metabolites was calculated by R package Limma (3.56.2). P value was calculated by R package stats (4.3.1). Specifically, the normal distribution test inner groups used Shapiro-Wilk Normality Test ($P > 0.05$, passed). Homogeneity of variance across groups was used Levene's test in R package car (3.1-2), Student's t-test (Levene's test, $P > 0.05$), or Welch's t-test (Levene's test, $P < 0.05$) used for calculating the p-value between two groups for every single metabolite. The t-test p value < 0.05 is considered statistically significant. Metabolism pathway enrichment used the algorithm from the R package MetaboAnalystR (4.0.0), which used the reference database KEGG (Oct. 2019). Pathway results and relative intensity data of the two groups were visualized by R package ggplot2 (3.4.2), ggrepel (0.9.3), and ggsignif (0.6.4). DEGs between groups, all genes' Log2FC and adjust p-value and PCA were analyzed by R package DESeq2 (1.40.2). GO and KEGG enrichment, GSEA analysis used R package clusterProfiler (4.9.1.001). PCA plot was performed by ggplot2 and ggrepel. Dot plot of GO and KEGG enrichment was performed by clusterProfiler. GSEA analysis was performed by R package GseaVis (0.0.8) (https://github.com/junjunlab/GseaVis).

Western blot. The cell treatment and grouping remained consistent with previous sections. Total proteins were extracted using a Cell lysis buffer for Western and IP (Beyotime, China) containing 1 mM protease inhibitor. Protein concentrations were determined using a BCA kit. Equal amounts of protein (20 μg) were loaded onto 8% and 4-20% sodium dodecyl sulfate polyacrylamide gel electrophoresis (SDS-PAGE) gels, followed by transfer to polyvinylidene fluoride (PVDF) membranes. The membranes were incubated with primary antibodies against FASN (Proteintech, China), mTOR (Proteintech, China), Phospho-mTOR (Proteintech, China), SREBP1 (Abcam, Britain), GPX4 (Santa Cruz, USA), SCD1 (Santa Cruz, USA), β-actin (Proteintech,

China) overnight at 4 °C. Following this, the membranes were washed in Tris-Buffered Saline (TBS) with Tween 20 (TBST) for 30 minutes and incubated with horseradish peroxidase (HRP) coupled secondary antibodies for 1 hour at room temperature. The ECL detection system was used to visualize target protein signals, with β -actin serving as a protein loading control.

Cellular uptake of nanomaterials and biodistribution *in vivo*. Cal-27 cells were inoculated into Petri dishes and co-cultured with IR-780 labeled *ER*-Cu_1SAZyme monoatomic materials (20 μg mL-1) for durations ranging from 0 to 12 h. Cellular uptake of *ER*-Cu_1SAZyme was observed by laser confocal microscope. For evaluating in vivo biodistribution, IR-780 labeled *ER*-Cu_1SAZyme was administered into tumor bearing mice *via* tail vein injection. At various time points post-injection (1, 2, 4, 8, 12, 24 and 48 h), fluorescence distribution within the mice was monitored using a multimodal animal *in vivo* imaging system (Boluteng, China). After the 48-hour time point, the animals were euthanized, and tissues including heart, liver, spleen, lung, kidney and tumor were harvested and examined using the imaging system.

***In vivo* antitumor experiment.** The *in vivo* anti-tumor efficacy was assessed using a subcutaneous tumor in nude mice. Twenty BALB/c-nu mice were randomly divided into 4 groups, each comprising 5 mice. Cal-27 cells were resuspended in PBS and mixed with a matrix (Corning, USA) adhesive in a 1:1 ratio. The cell suspension was injected subcutaneously on the right flank of the mice. Once the tumor volume reached approximately 100-200mm^3, drug administration *via* the caudal vein was initiated, with injections given every other day for a total of 8 doses. For the assessment of anti-tumor effects, the vertical diameter of tumor was measured by using a vernier caliper every two days, and the body weight of the mice was monitored. The tumor volume was calculated using the formula: $V\ (mm^3) = (length \times width^2) / 2$. Upon completion of the dosing cycles, the tumors, as well as the hearts, livers, spleens, lungs and kidneys of the mice, were excised for H&E staining and immunohistochemical detection. Concurrently, blood serum was collected from the mice to determine the levels of creatinine (Cr), glutamic pyruvic transaminase (GPT), and glutamic oxaloacetic transaminase (GOT) using respective detection kits from Biosharp (China) and Solarbio (China).

Biosafety assessment. The toxicity of *ER*-Cu_1SAZyme towards human normal somatic cells was gauged using a CCK-8 cell proliferation assay kit. Human renal tubular epithelial HK-2 cells were plated in 96-well plates at the density of 5000 cells per well and incubated for 24 h. They were then exposed to a range of *ER*-Cu_1SAZyme concentrations (0, 1, 2, 4, 8, 16, 32, 64, 128, 256 μg mL^{-1}) for both 24 h and 48 h. The culture medium was subsequently removed, and the wells were washed with PBS before the addition of 10 μL of CCK-8 solution and 90 μL of fresh medium, followed by a 2-hour incubation at 37 °C. The absorbance at

450nm was recorded using a microplate reader, and cell survival rates were calculated as previously described. To assess the hemocompatibility of *ER*-Cu_1SAZyme, a hemolysis test was conducted. Mouse blood was centrifuged at 3000 rpm and washed with PBS to isolate pure red blood cells. A 4% red blood cell suspension (0.5ml) was mixed with various concentrations of *ER*-Cu_1SAZyme (0, 1, 2, 4, 8, 16, 32, 64, 128, 256 μg mL-1) nanomaterials in PBS (0.5ml) and incubated at 37 °C for 8 h. Deionized water served as a positive control, while normal saline acted as a negative control. After centrifugation, the supernatant's absorbance at 545 nm was determined using a UV VIS spectrometer. In addition, liver and kidney functions in mice from the in vivo anti-tumor experiments were evaluated.

Statistical analysis. All data are expressed as the mean ± standard error of the mean. $p < 0.05$ was considered to be statistically significant. Except for the special instructions, asterisks (*) denote statistical significance between bars (*$p < 0.05$, **$p < 0.01$, ***$p < 0.001$) conducted using a t-test.

Reporting summary. Further information on research design is available in the Nature Portfolio Reporting Summary linked to this article.

Data availability. All data supporting the findings of this study are available within the Article, Supplementary Information or Source Data file.

Supplementary Figures

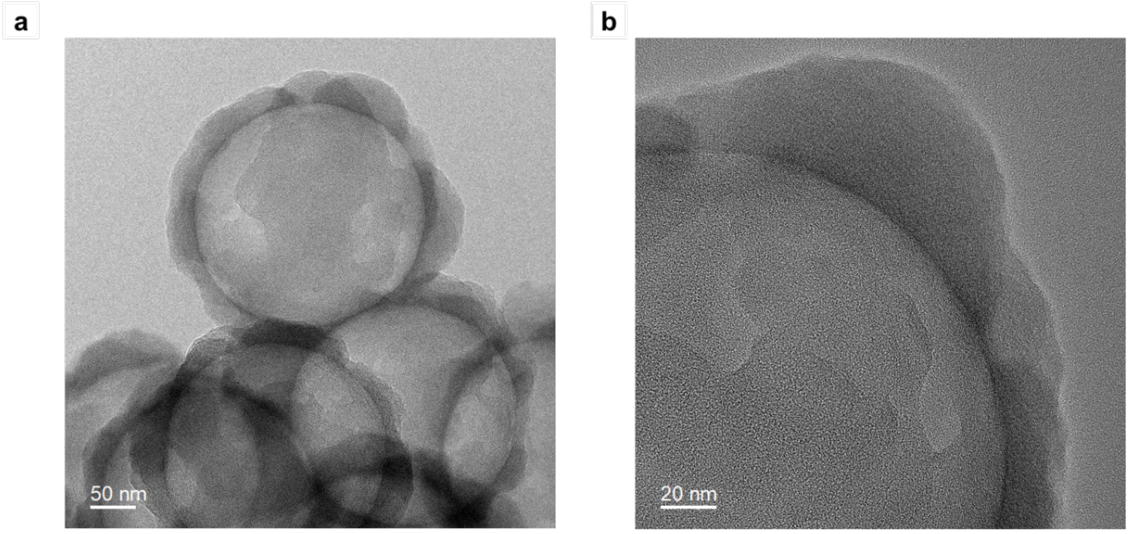

Figure S1. (**a-b**) TEM images of the synthesized *ER*-Cu_1SAZyme.

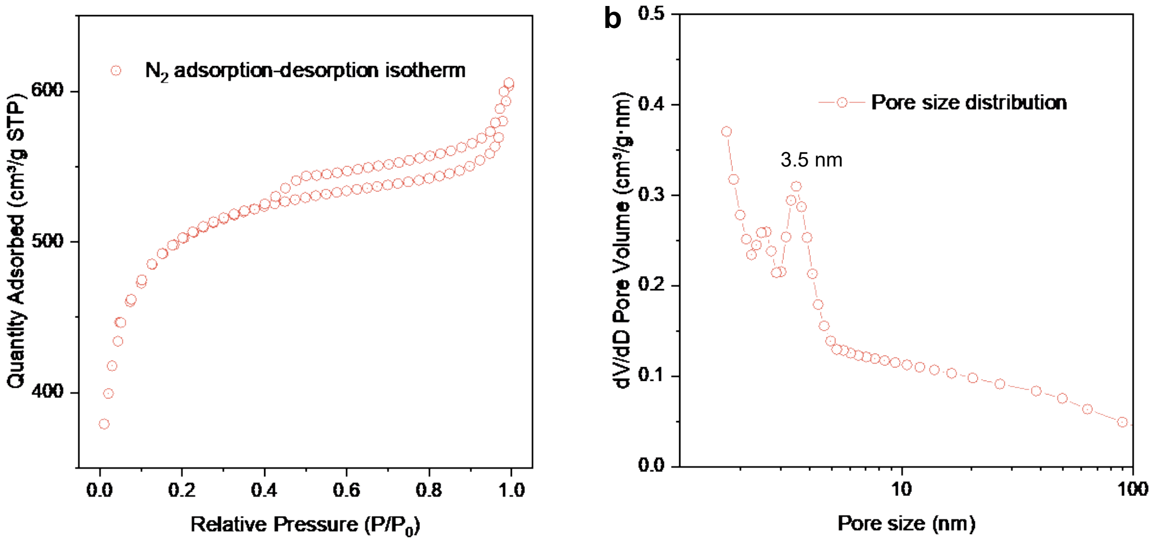

Figure S2. (**a**) N_2 adsorption and desorption isotherms of the *ER*-Cu_1SAZyme. (**b**) Corresponding pore-size distribution.

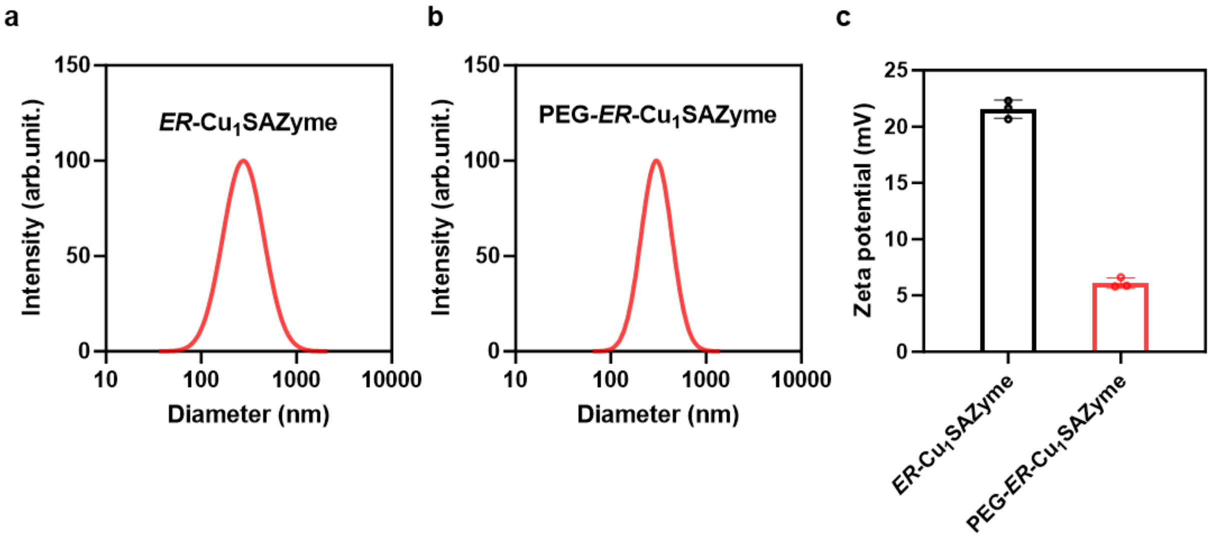

Figure S3. Hydrated particle size and zeta potential of *ER*-Cu_1SAZyme.

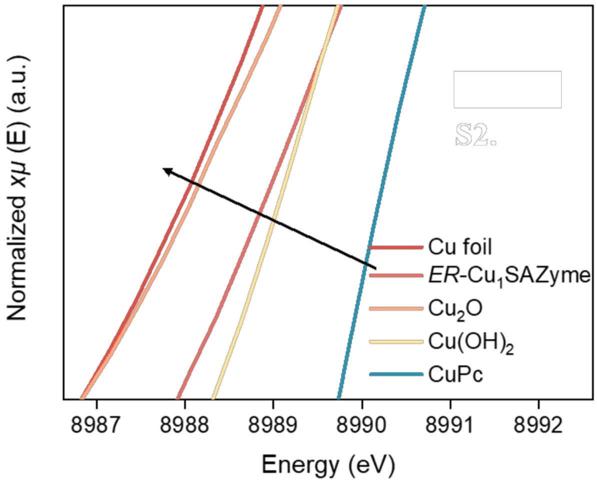

Figure S4. Corresponding enlarged Cu *K*-edge XANES spectra.

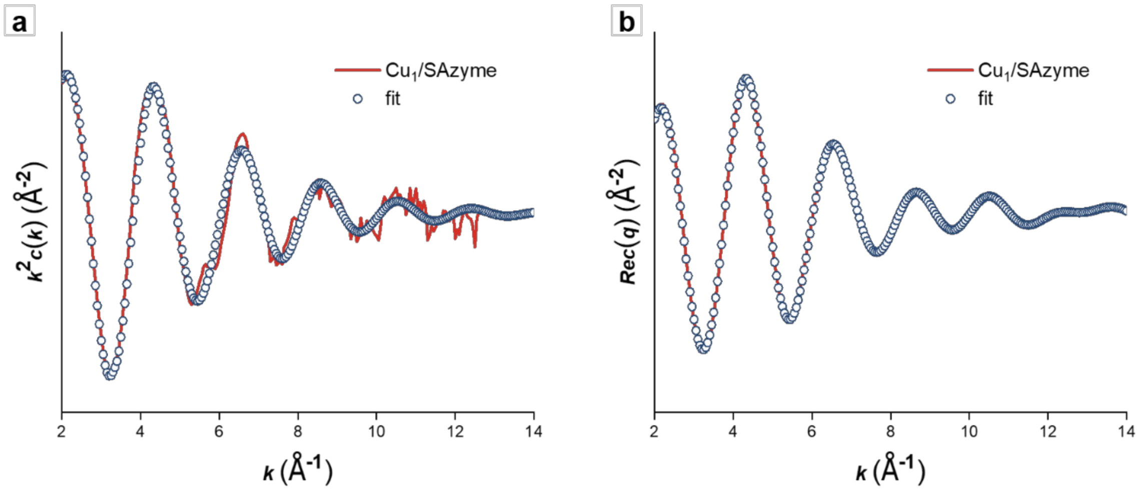

Figure S5. EXAFS fitting curves of *ER*-Cu_1SAZyme. (a) the FT-EXAFS fitting curve at Cu K-edge and (b) q space fitting curve. The structural parameters are listed in Table S2.

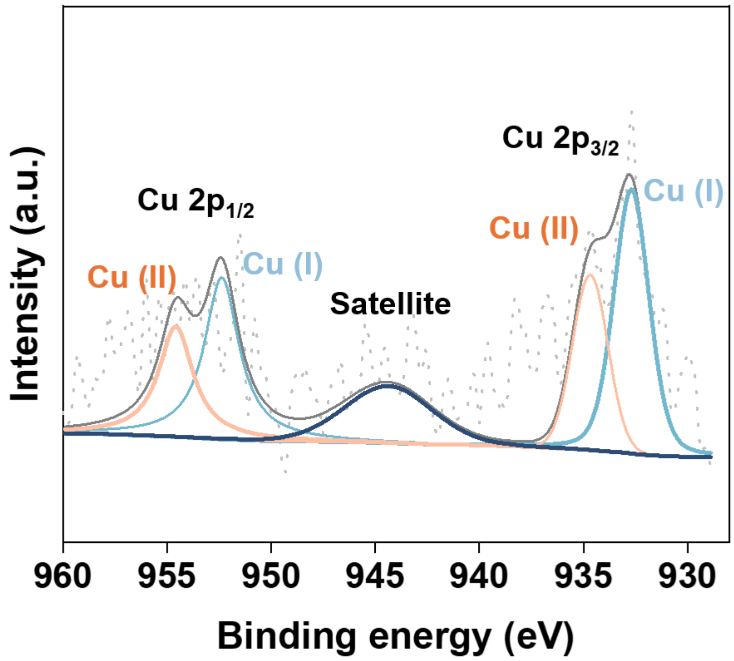

Figure S6. Cu 2p XPS spectra of the *ER*-Cu_1SAZyme.

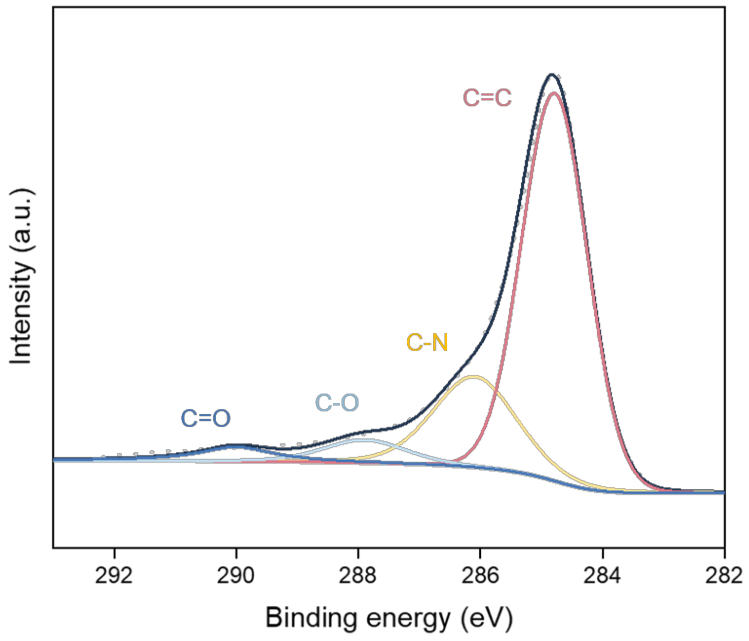

Figure S7. C 1s XPS spectra of *ER*-Cu₁SAZyme.

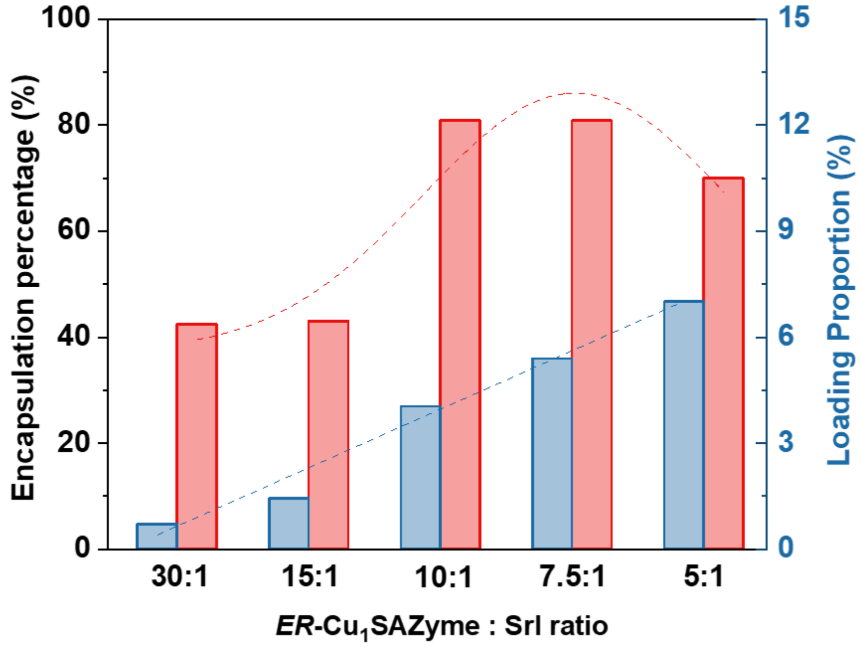

Figure S8. The Encapsulation percentage and loading proportion of *ER*-Cu₁SAZyme: SrI.

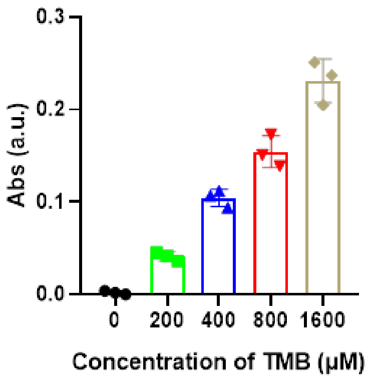

Figure S9. POD-like activity of Cu_1/SAzyme by TMB chromogenic reaction. The concentration of TMB ranged from 0 to 1600 μM.

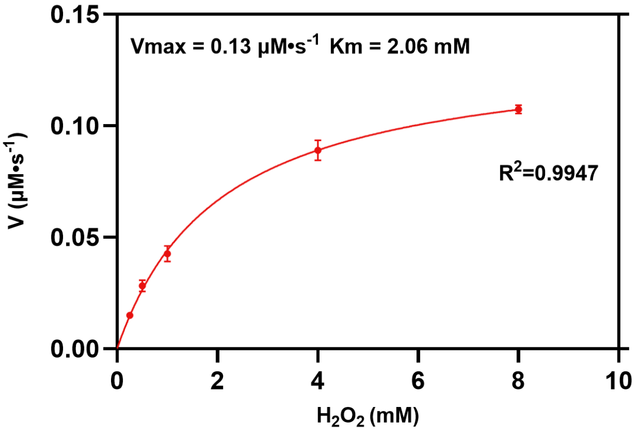

Figure S10. Steady-state kinetic analysis of the peroxidase-like activity of *ER*-Cu_1SAZyme using H_2O_2 as substrate. The reaction rates were measured at varying H_2O_2 concentrations (0.25, 0.5, 1, 4, and 8 mM), and the data were fitted using the Michaelis-Menten model to obtain kinetic parameters.

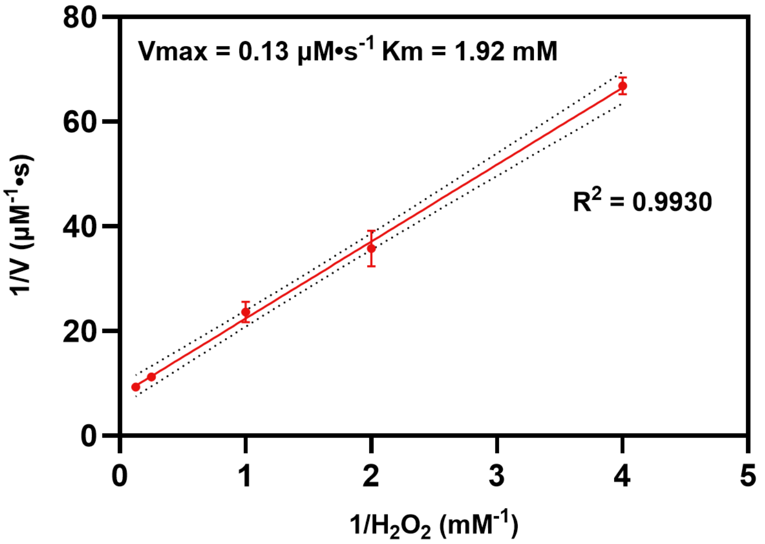

Figure S11. Lineweaver-Burk plot of the peroxidase-like activity of *ER*-Cu_1SAZyme using H_2O_2 as substrate. Reaction rates were measured at varying H_2O_2 concentrations (0.25, 0.5, 1, 4, and 8 mM), and the data were fitted using the Lineweaver-Burk method to derive steady-state kinetic parameters (Km and Vmax).

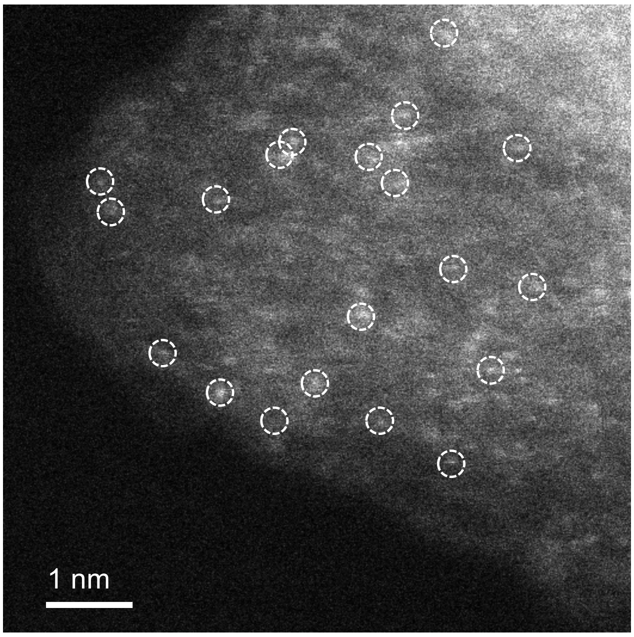

Figure S12. Atomic-resolution HAADF-STEM images of the non-edge Cu_1SAZyme.

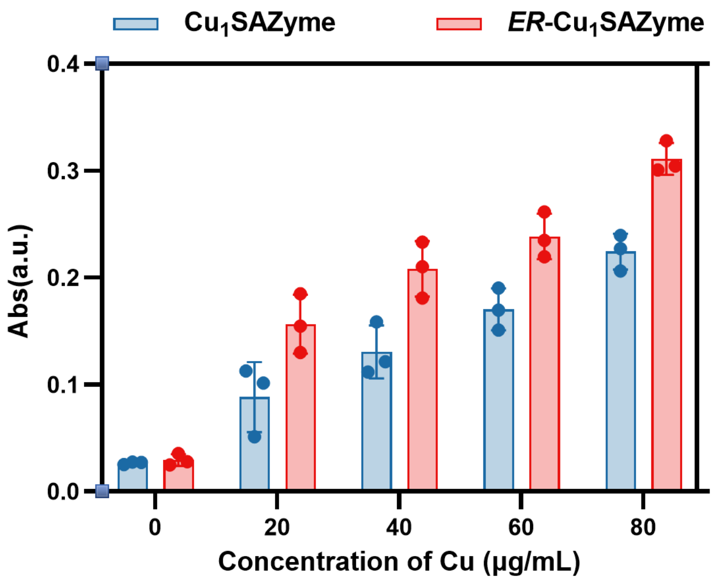

Figure S13. Comparison of POD activity between *ER*-Cu₁SAZyme and non-edge-enriched Cu₁SAZyme across a gradient of Cu concentrations (0, 20, 40, 60, 80 μg/mL). POD activity was evaluated using a standard TMB oxidation assay.

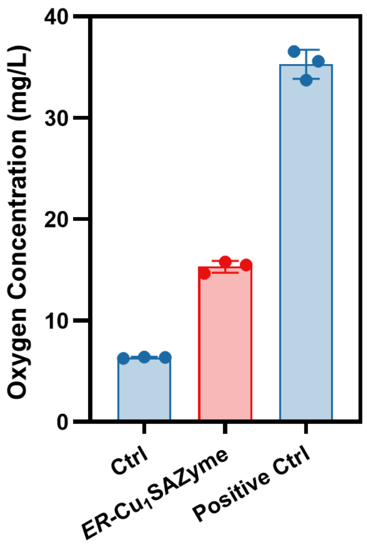

Figure S14. Catalase-like activity assay based on oxygen generation. Oxygen concentration (mg/L) was measured using a dissolved oxygen meter after treatment with control, *ER*-Cu₁SAZyme, and positive control.

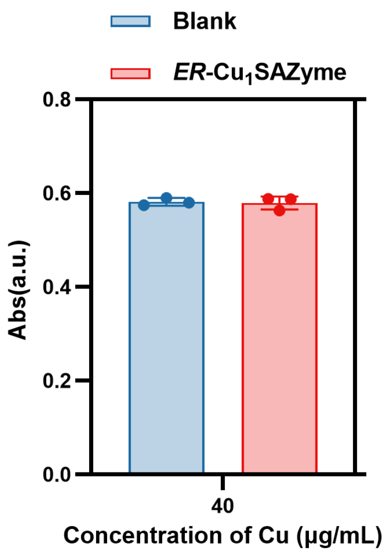

Figure S15. Superoxide dismutase (SOD)-like activity assay of *ER*-Cu_1SAZyme at a Cu concentration of 40 μg/mL. The assay was performed using the nitroblue tetrazolium (NBT) photoreduction method, and absorbance was measured to evaluate inhibition of superoxide-mediated NBT reduction. The x-axis represents Cu concentration (μg/mL), and the y-axis indicates absorbance.

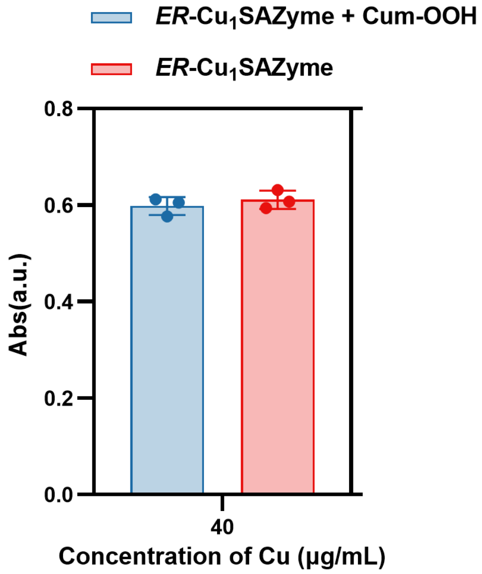

Figure S16. Glutathione peroxidase (GPx)-like activity assay of *ER*-Cu_1SAZyme with or without Cum-OOH as substrate. The assay was performed using a Total Glutathione Peroxidase Assay Kit with DTNB, and absorbance was measured at 412 nm. The Cu concentration was fixed at 40 μg/mL. Two groups were tested: *ER*-Cu_1SAZyme alone and *ER*-Cu_1SAZyme + Cum-OOH. The x-axis represents Cu concentration (μg/mL), and the y-axis indicates absorbance.

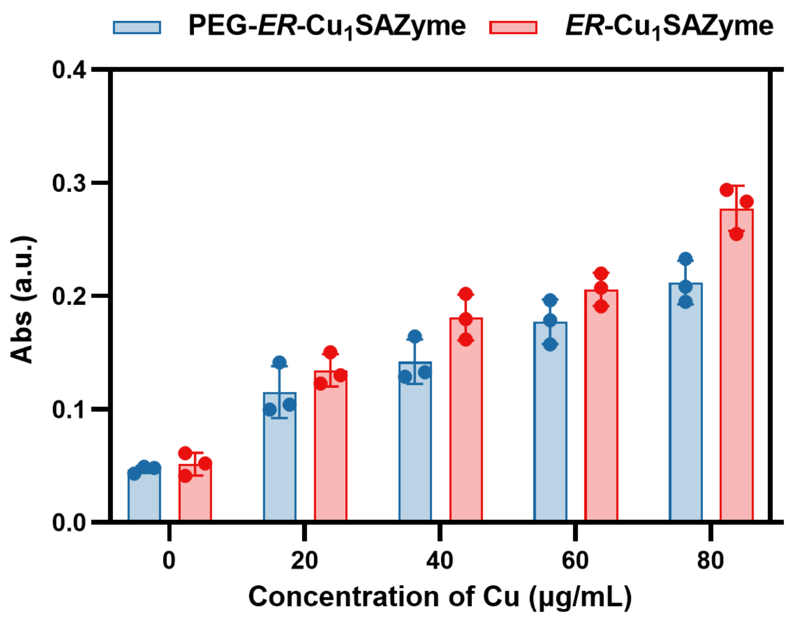

Figure S17. Comparison of peroxidase-like (POD) activity between *ER*-Cu_1SAZyme and PEG-*ER*-Cu_1SAZyme at varying Cu concentrations (0, 20, 40, 60, 80 μg/mL). The catalytic activity was assessed using a standard TMB oxidation assay.

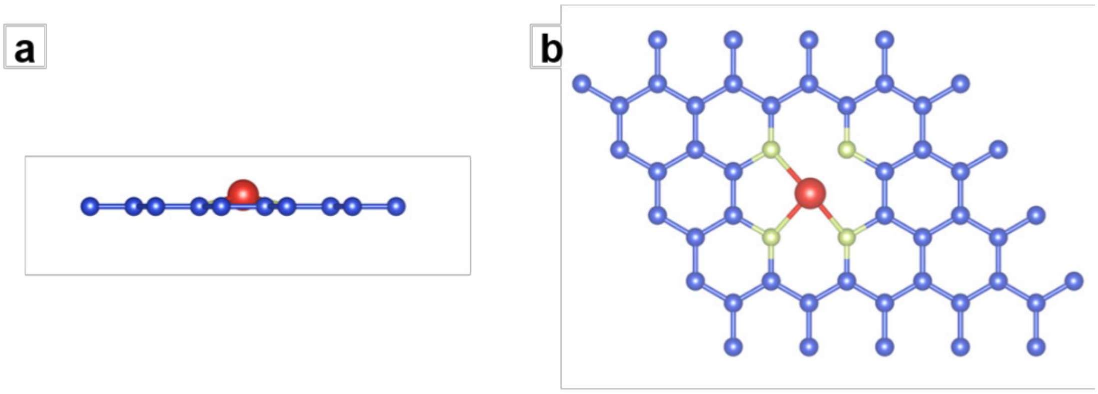

Figure S18. Corresponding atomic coordination structure model of *ER*-Cu_1SAZyme.

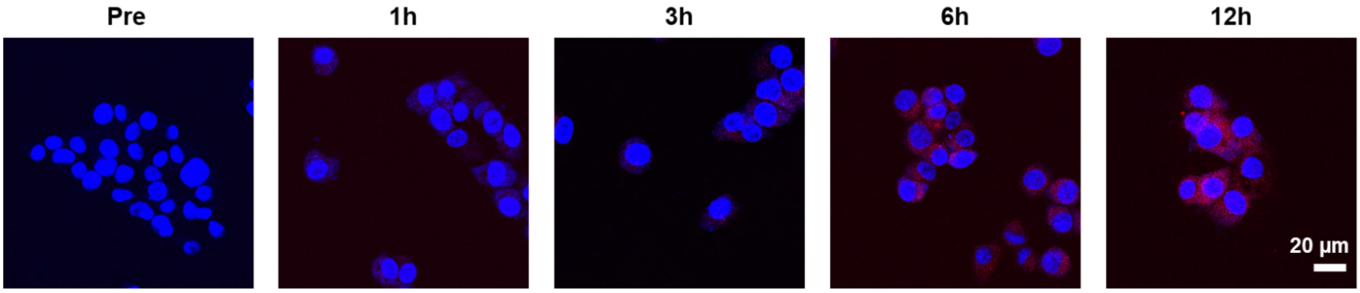

Figure S19. Confocal microscopy images of Cal-27 cells after incubation with RF/*ER*-Cu_1SAZyme (red: RF/*ER*-Cu_1SAZyme, blue: Hoechst).

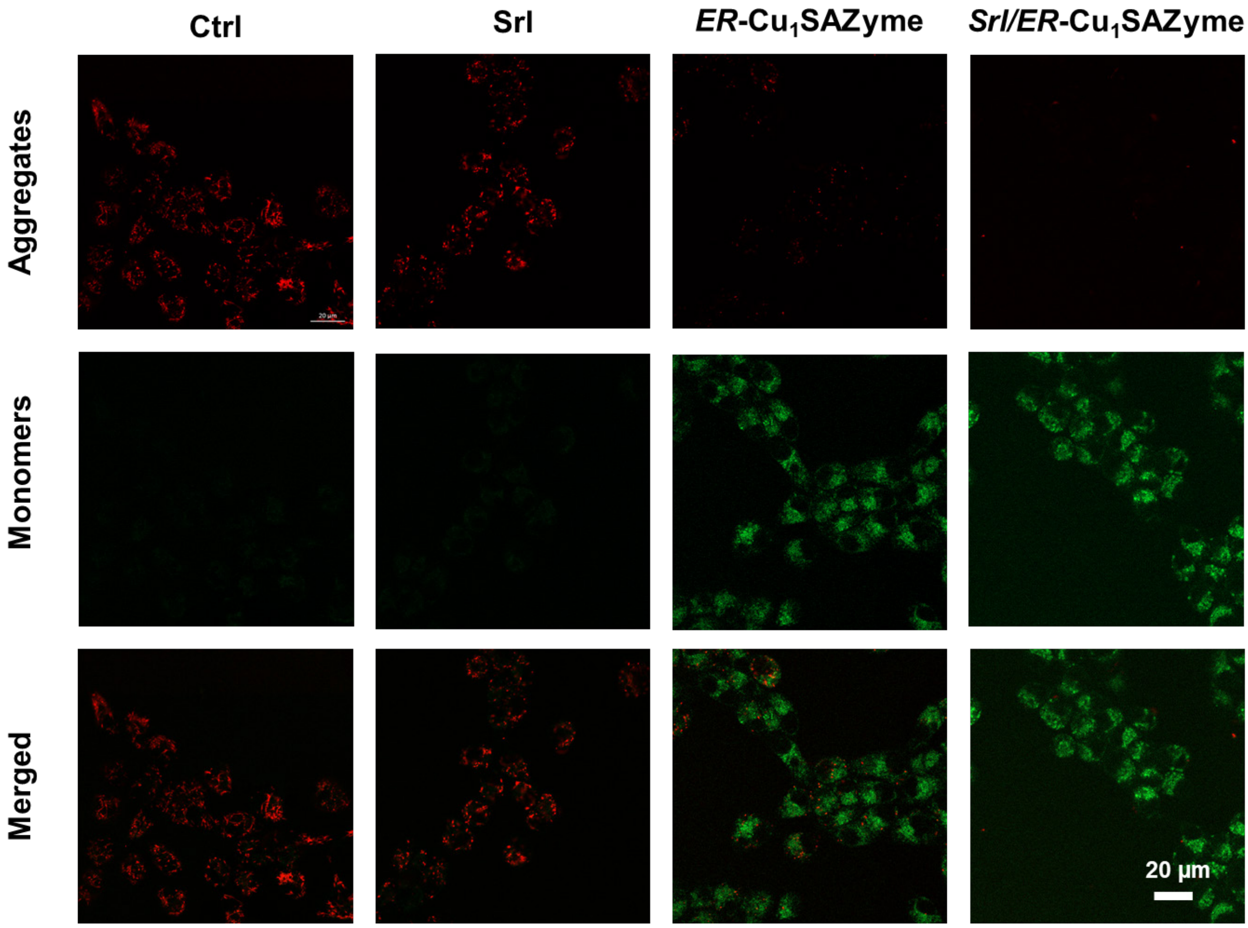

Figure S20. JC-1 analysis of cells as a measure of mitochondrial depolarization. The color shows JC-1 aggregates (red) in mitochondria and JC-1 monomers (green) in cytosol.

β-actin

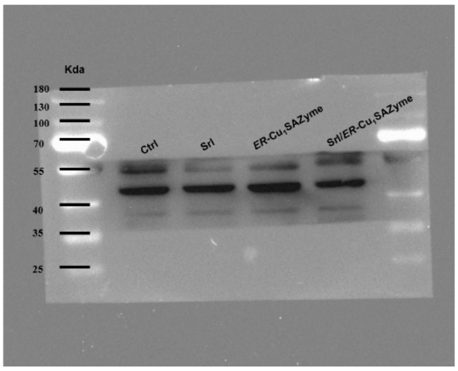

GPX4

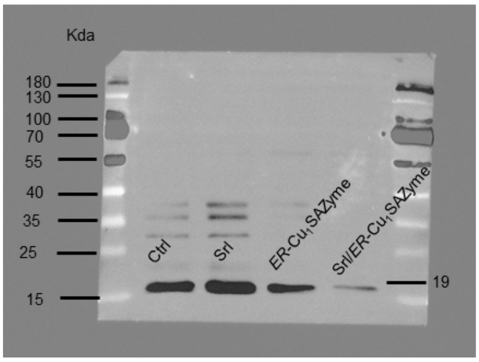

Figure S21. Unprocessed western blot analysis of GPX4 expression in Cal-27 cells treated with Srl/*ER*-Cu_1SAZyme.

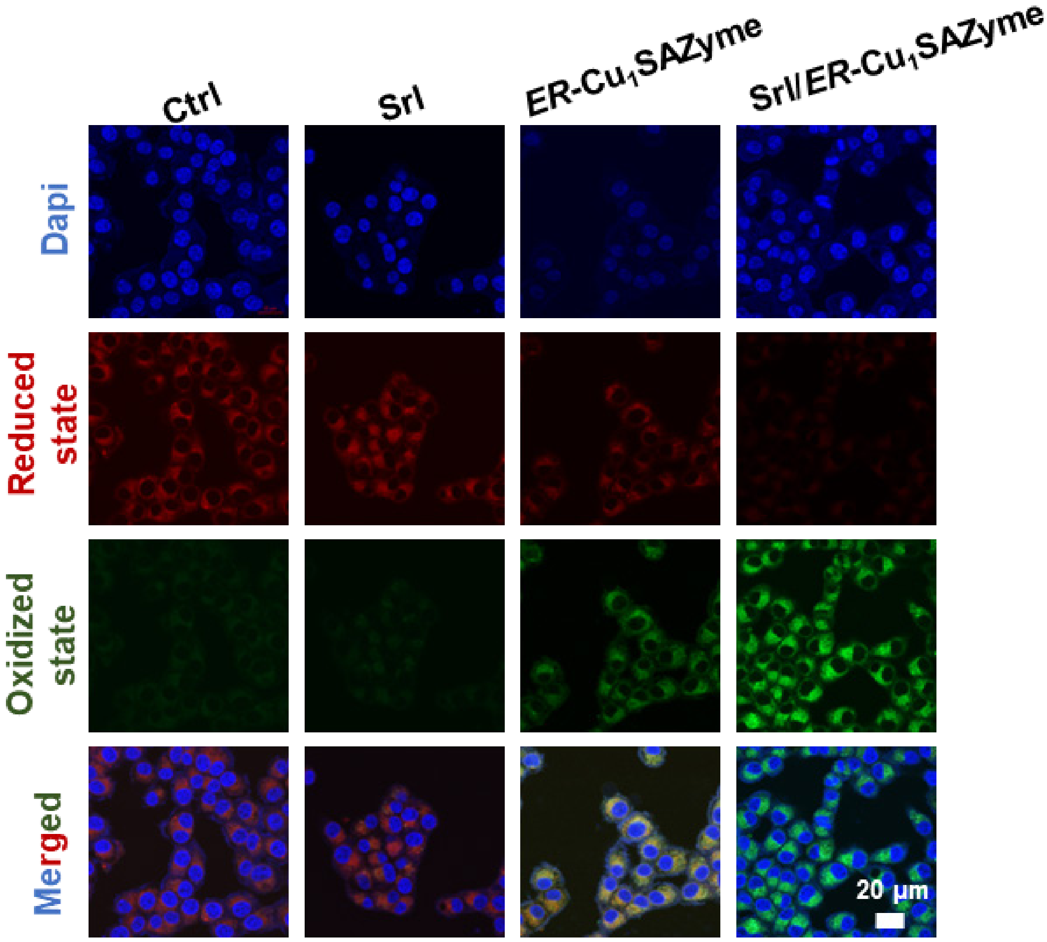

Figure S22. Lipid peroxidation levels measured by BODIPY-C11 staining in Cal-27 cells after Srl/*ER*-Cu_1SAZyme treatment.

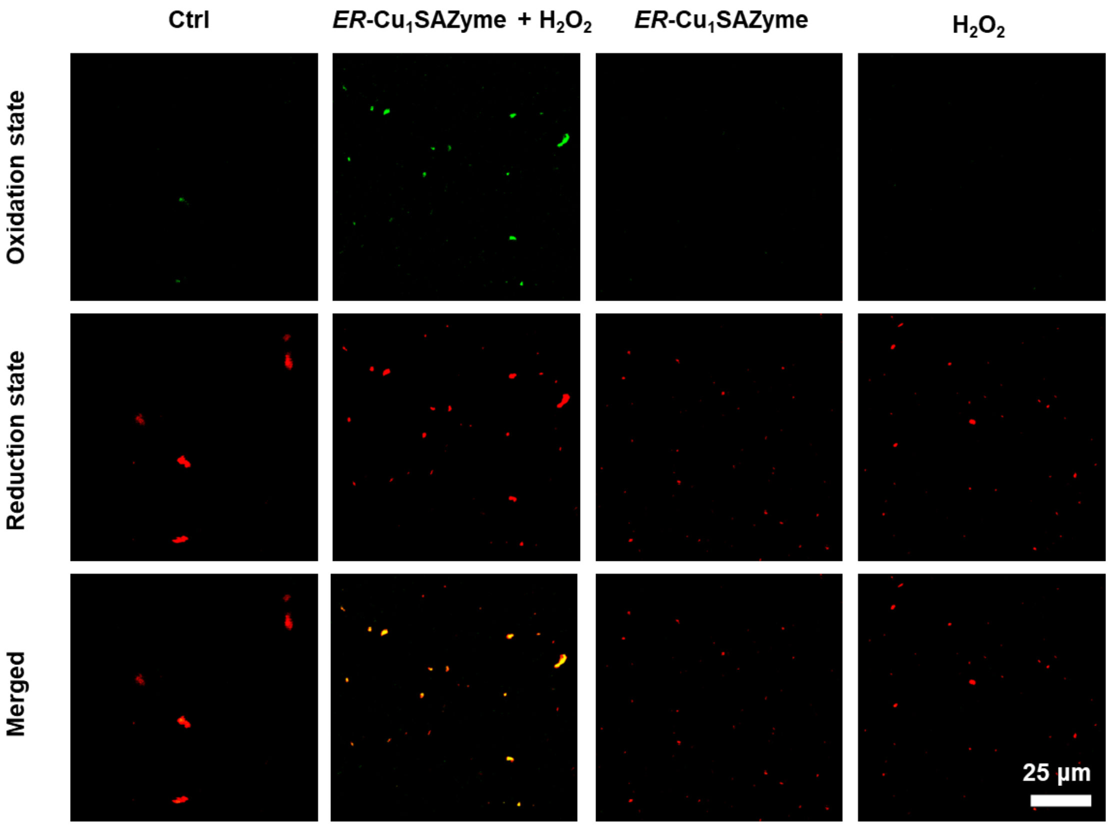

Figure S23. Confocal fluorescence imaging of lipid peroxidation in a model liposome system using BODIPY 581/591 C11 as a reporter. Liposomes were prepared *via* thin-film hydration with lecithin and extruded to form uniform vesicles. Samples were treated with *ER*-Cu_1SAZyme, H_2O_2, or their combination, and fluorescence signals were recorded. Red fluorescence (~590 nm) indicates the reduced form of the probe, while green fluorescence (~510 nm) reflects oxidized BODIPY. Merge images show spatial overlap of red and green signals.

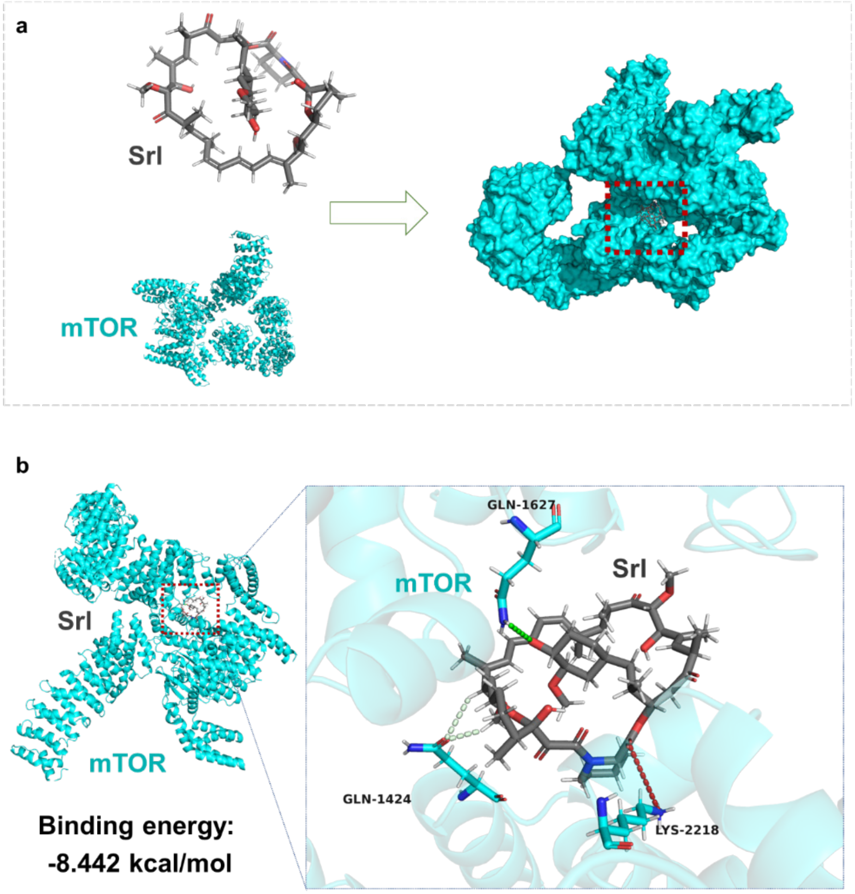

Figure S24. Molecular docking schematic. structural formulas of Srl and mTOR, with close-up views of the interaction sites.

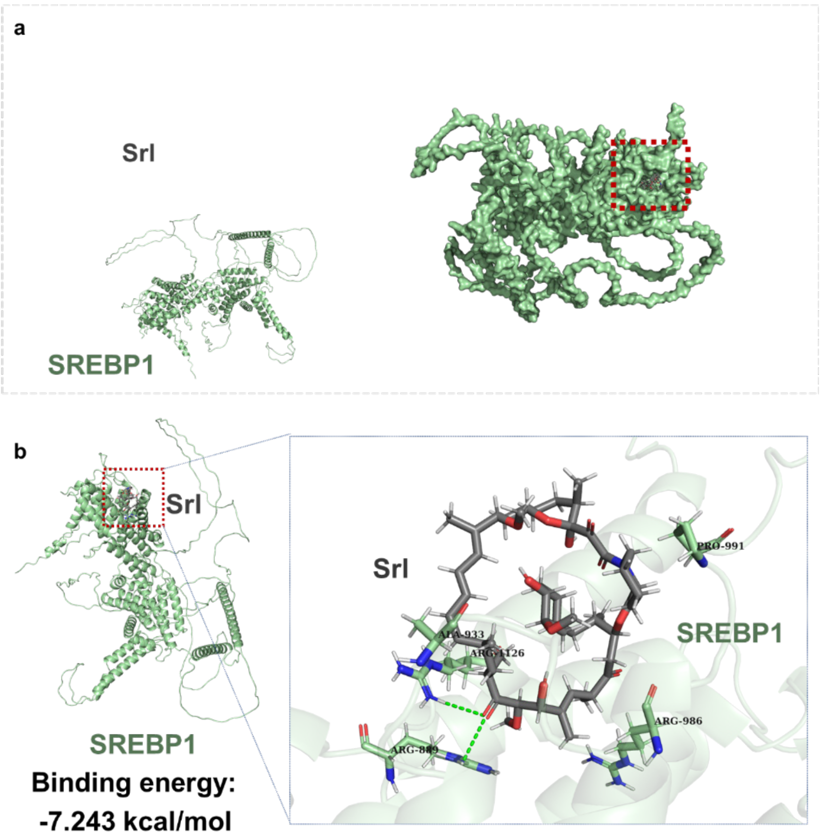

Figure S25. Molecular docking schematic. structural formulas of Srl and SREBP1, with close-up views of the interaction sites.

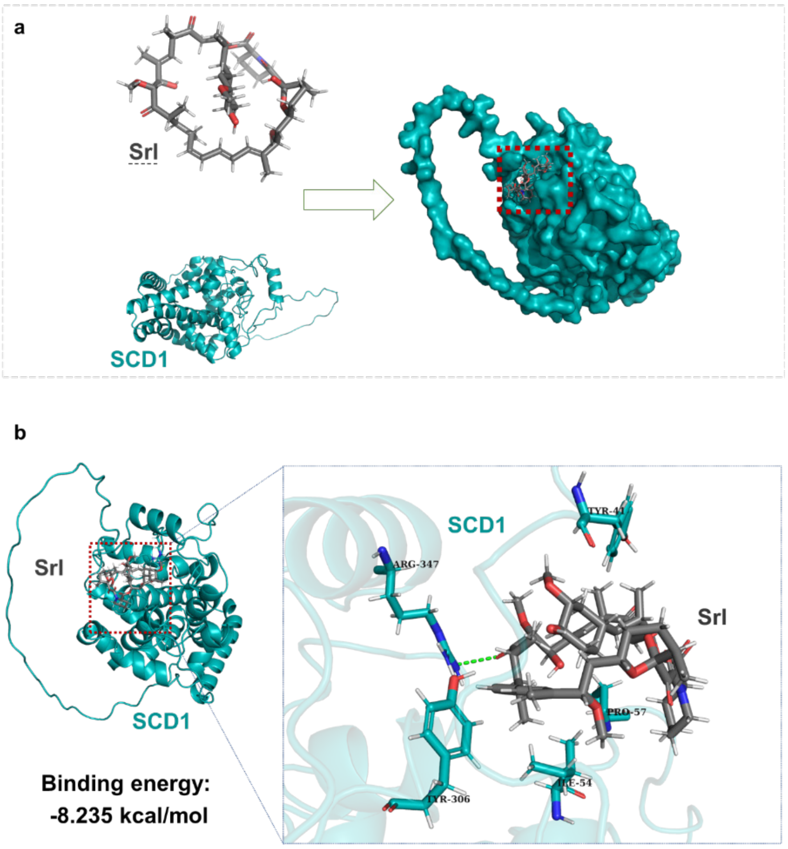

Figure S26. Molecular docking schematic. structural formulas of Srl and SCD1, with close-up views of the interaction sites.

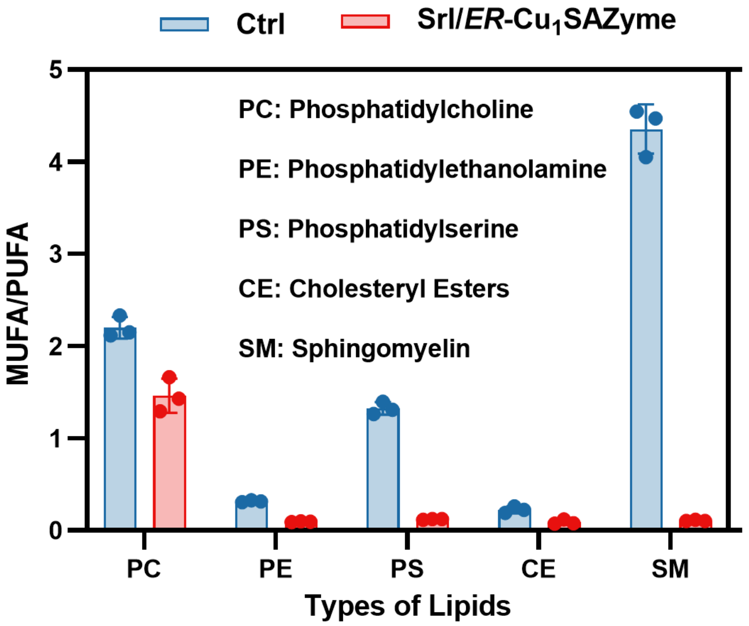

Figure S27. Targeted lipidomic analysis of membrane-associated lipid classes in Cal-27 cells using HPLC-MS/MS. The MUFA/PUFA ratio was quantified for five lipid categories-phosphatidylcholine (PC), phosphatidylethanolamine (PE), phosphatidylserine (PS), cholesteryl esters (CE), and sphingomyelin (SM)-under Ctrl and Srl/*ER*-Cu_1SAZyme treatment conditions.

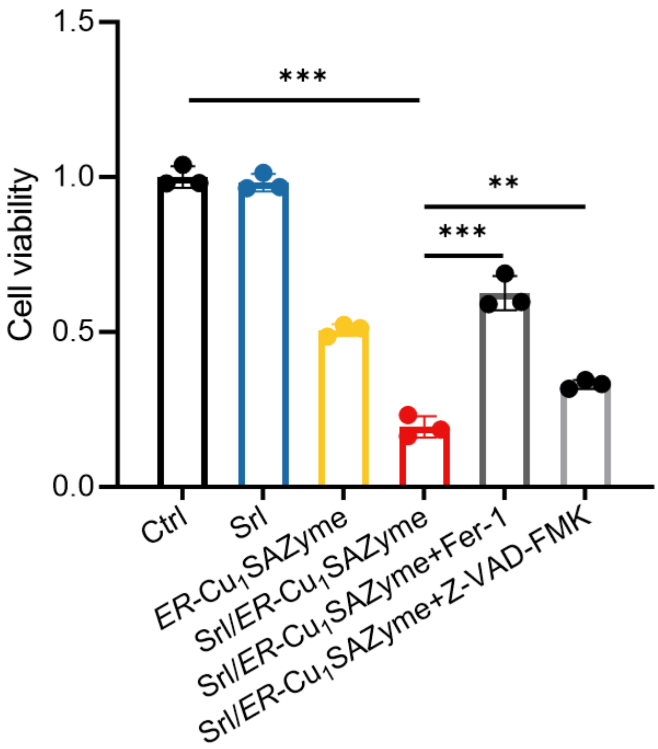

Figure S28. Cell *via*bility of Cal-27 cells under different treatment conditions.

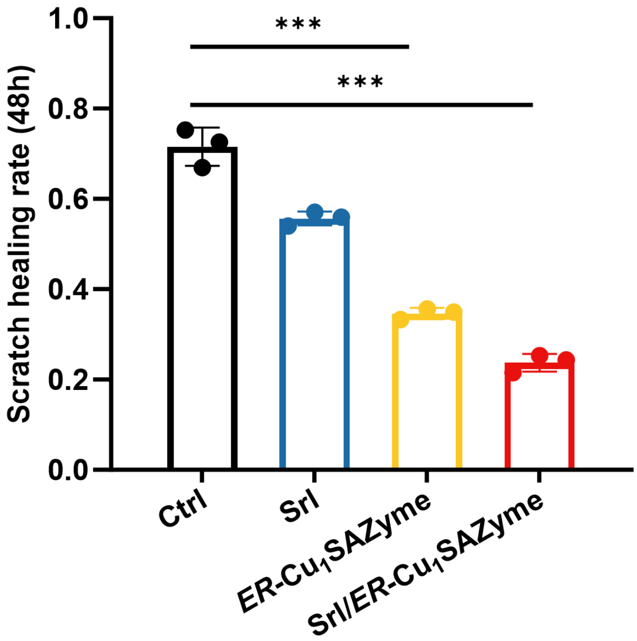

Figure S29. Scratch wound-healing assay showing the inhibitory effect of Srl/*ER*-Cu_1SAZyme on the migration of Cal-27 cells.

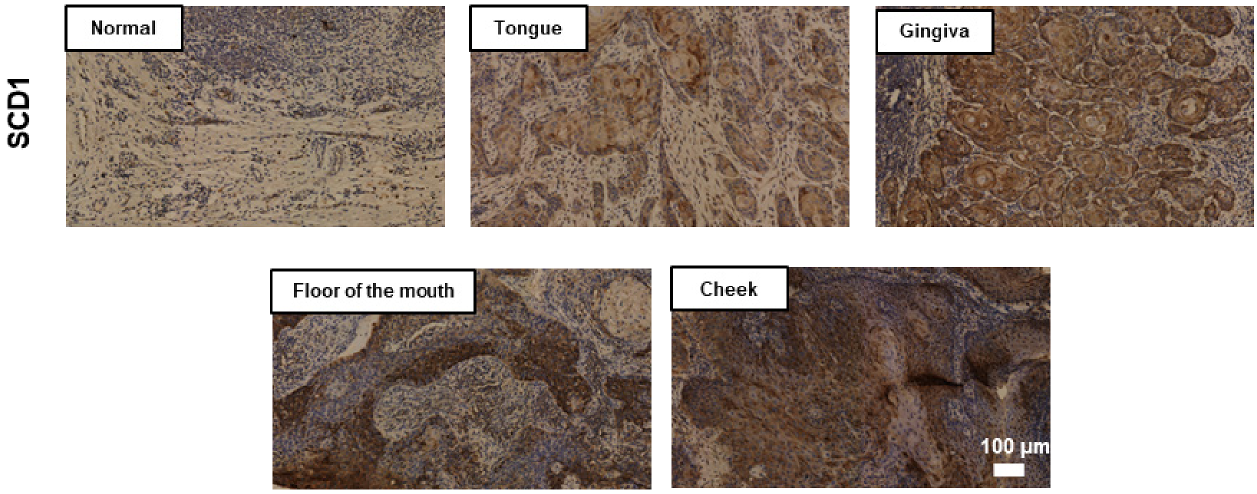

Figure S30. Representative immunohistochemical staining images of SCD1 in clinical samples.

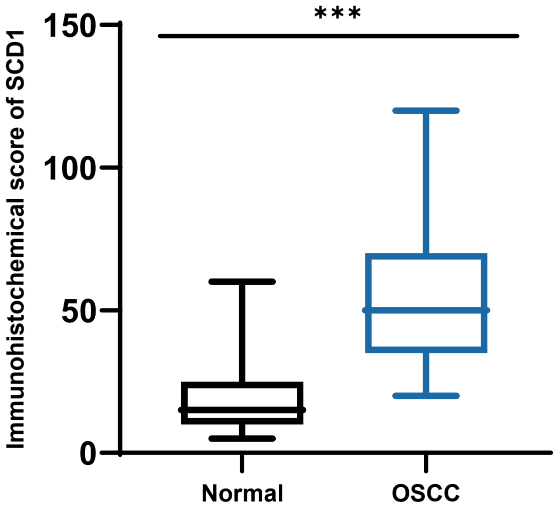

Figure S31. Immunohistochemical scores of SCD1.

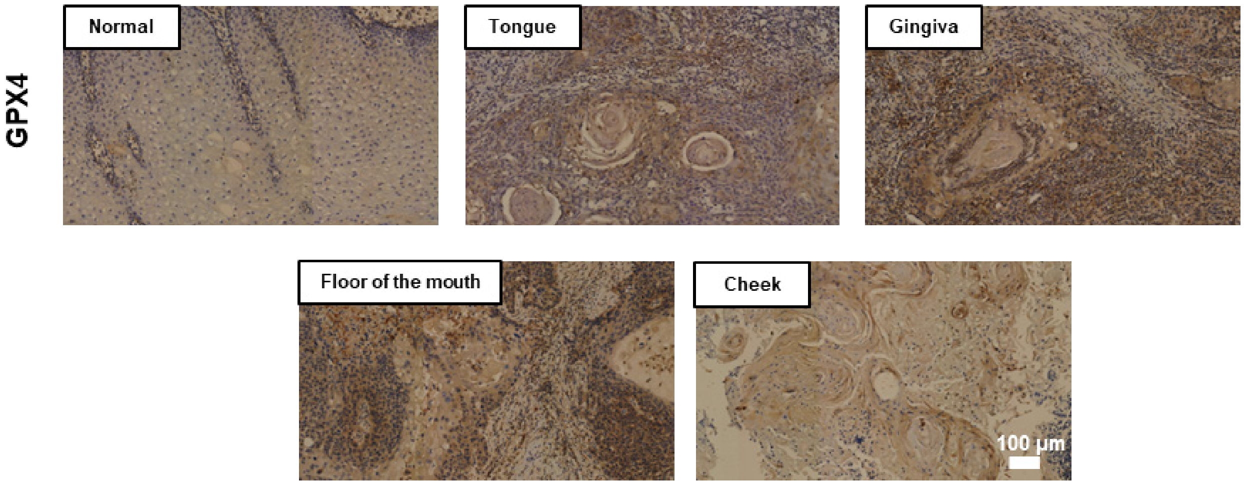

Figure S32. Representative immunohistochemical staining images of GPX4 in clinical samples.

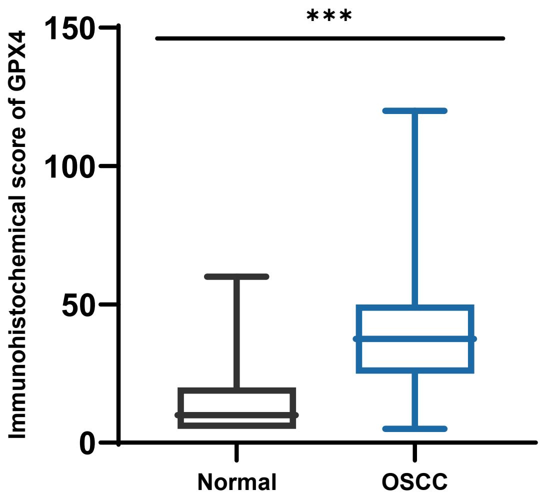

Figure S33. Immunohistochemical scores of GPX4.

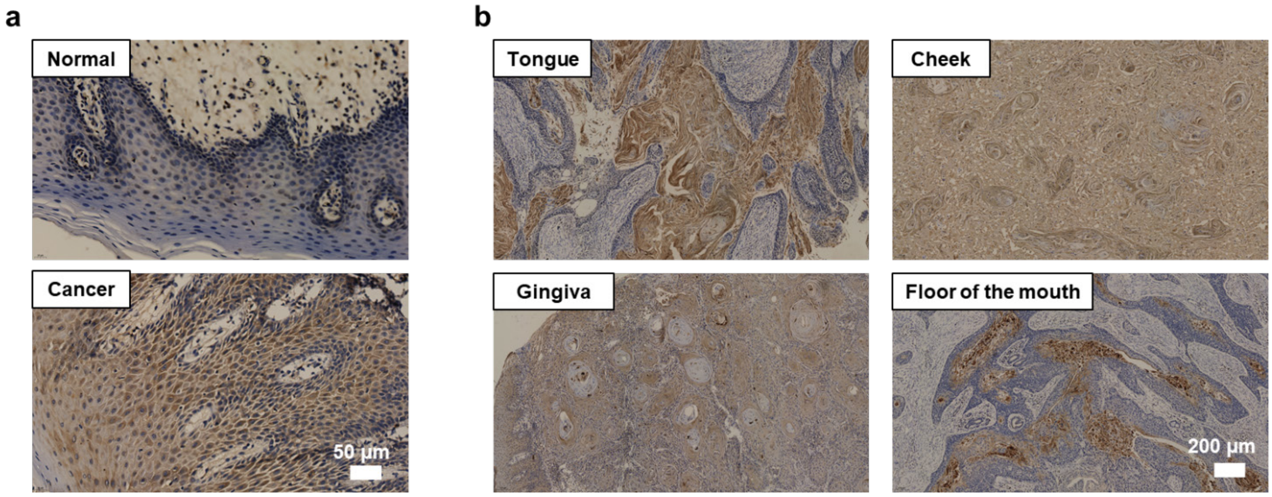

Figure S34. Representative immunohistochemistry images of mTOR in clinical patient samples. a. Normal and cancer tissues; b, Cancer tissues from the tongue, cheek, gingiva, and floor of the mouth.

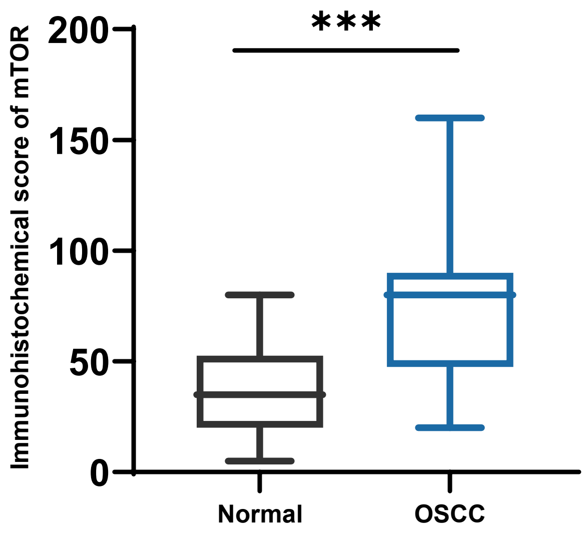

Figure S35. Immunohistochemical scores of mTOR.

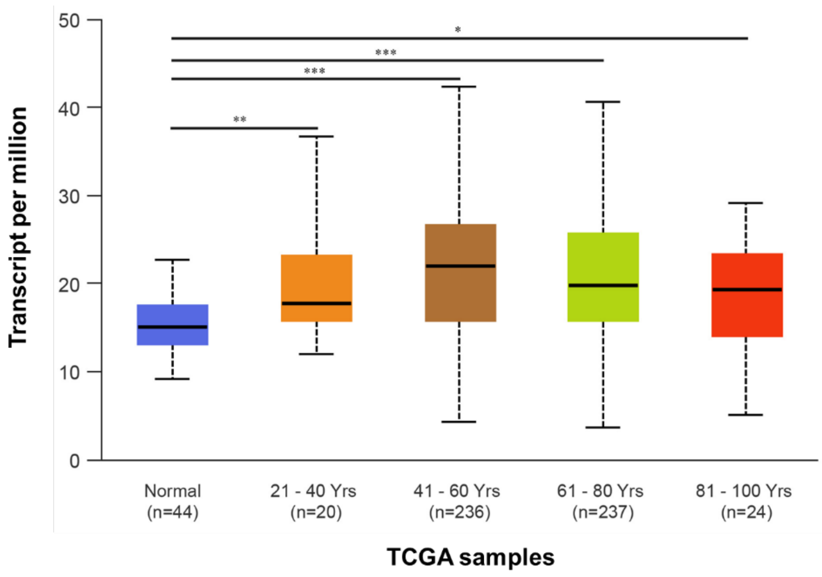

Figure S36. Expression of mTOR in HNSC based on patient's age.

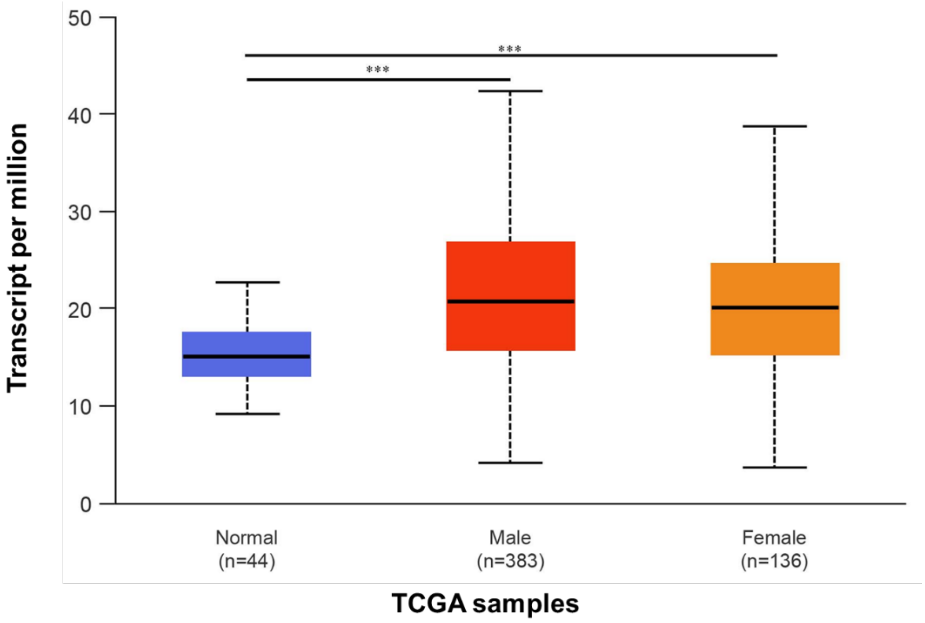

Figure S37. Expression of mTOR in HNSC based on patient’s gender.

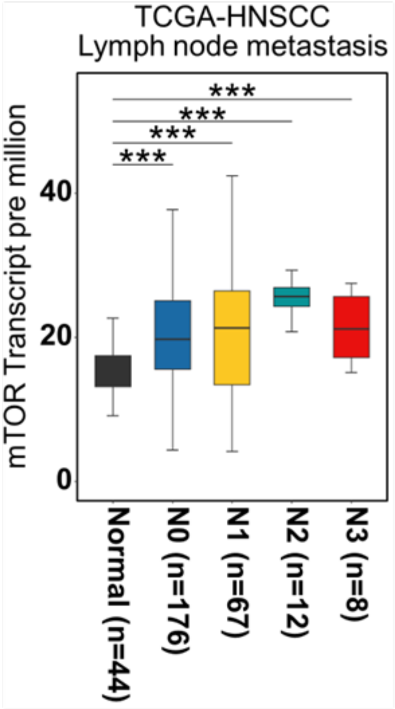

Figure S38. Expression of mTOR in HNSCC based on nodal metastasis status.

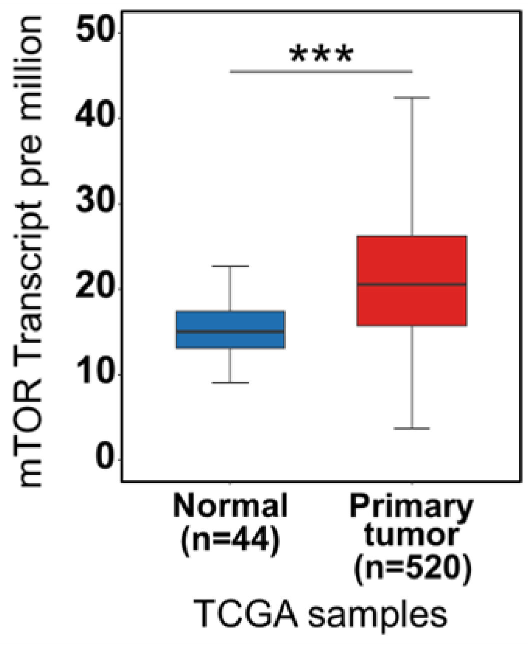

Figure S39. Expression of mTOR in HNSCC based on sample types.

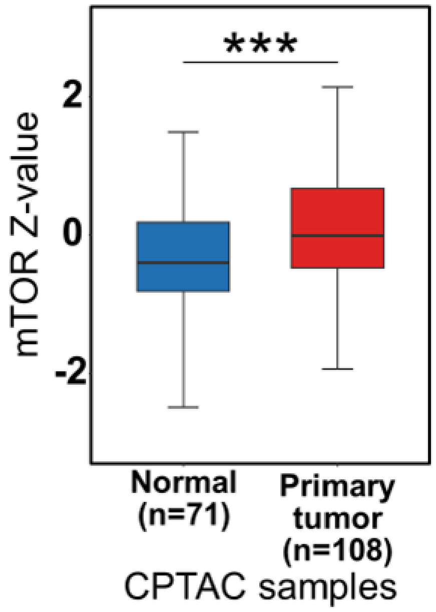

Figure S40. Protein expression of mTOR in HNSCC.

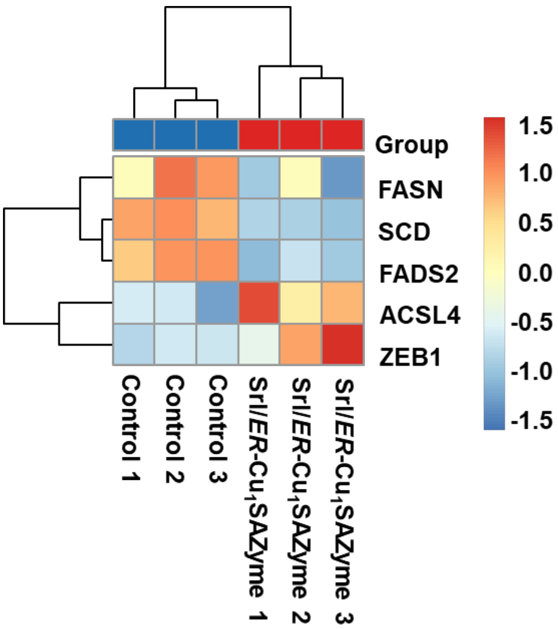

Figure S41. Heat map showing intracellular transcriptome regulation levels in Srl/*ER*-Cu_1SAZyme-treated Cal-27 cells. Blue, downregulated DEGs; red, upregulated DEGs.

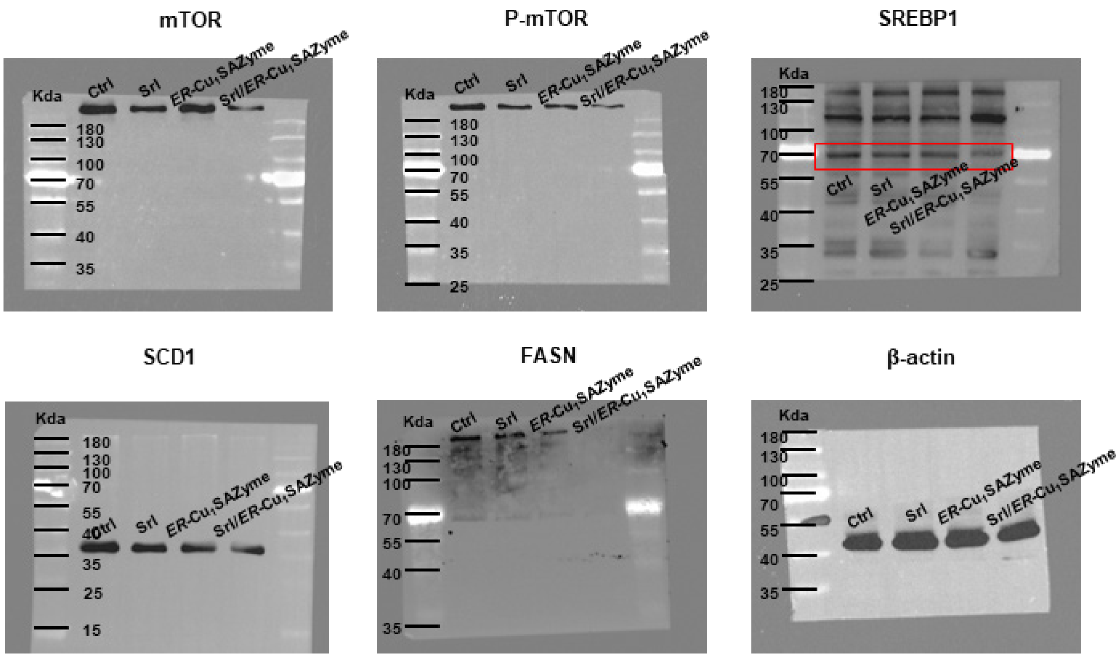

Figure S42. Unprocessed western blot analysis of mTOR, p-mTOR, m-SREBP1, SCD1 and FASN expression in Srl/*ER*-Cu_1SAZyme.

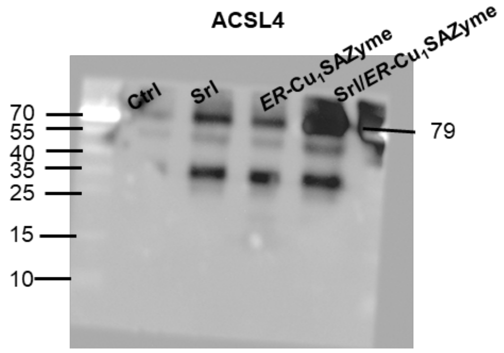

Figure S43. Unprocessed western blot analysis of ACSL4 expression in Srl/*ER*-Cu_1SAZyme.

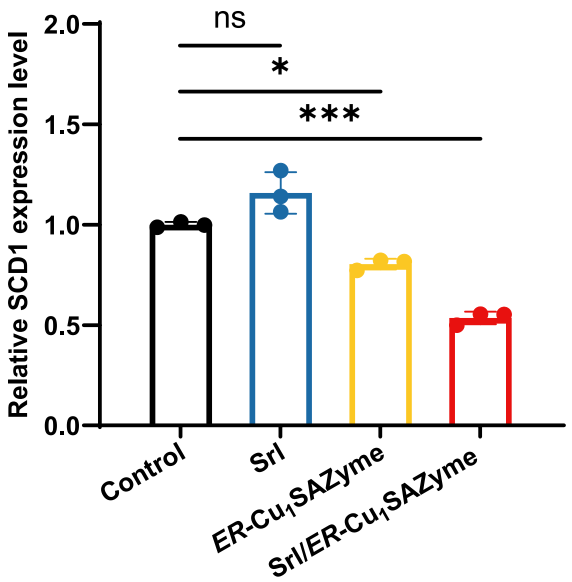

Figure S44. Quantification of SCD1 protein levels normalized to untreated controls and relative to β-actin as a loading control. Band intensities were analyzed using ImageJ software and presented as fold change relative to control.

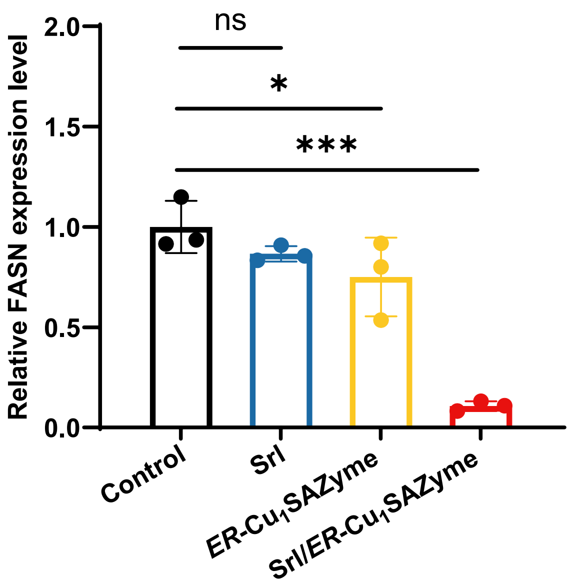

Figure S45. Quantification of FASN protein levels normalized to untreated controls and relative to β-actin as a loading control. Band intensities were analyzed using ImageJ software and presented as fold change relative to control.

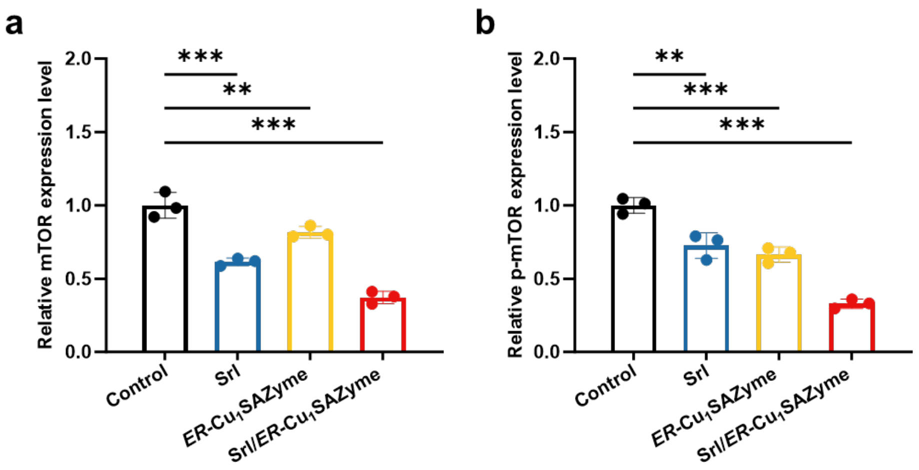

Figure S46. Quantification of mTOR protein levels normalized to untreated controls and relative to β-actin as a loading control. Band intensities were analyzed using ImageJ software and presented as fold change relative to control.

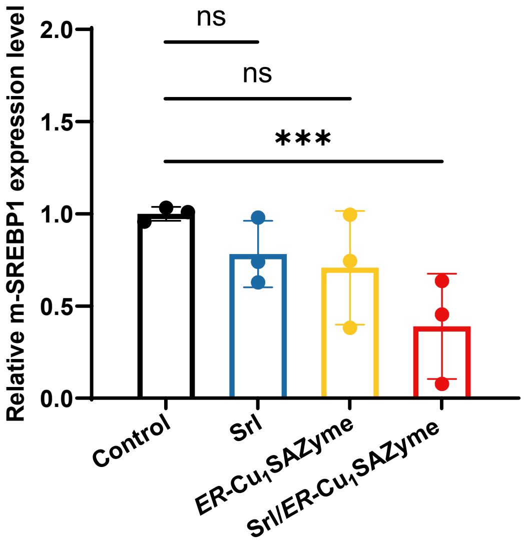

Figure S47. Quantification of SREBP1 protein levels normalized to untreated controls and relative to β-actin as a loading control. Band intensities were analyzed using ImageJ software and presented as fold change relative to control.

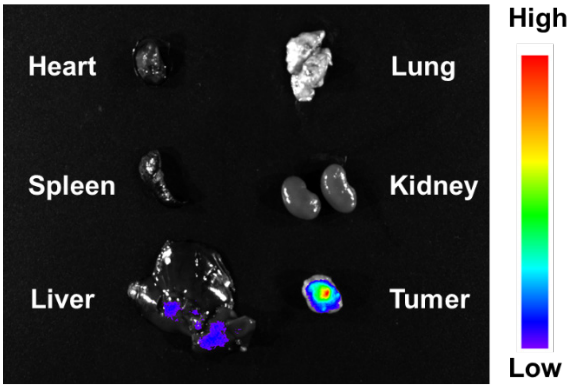

Figure S48. *Ex vivo* biodistribution of Srl/*ER*-Cu_1SAZyme in major organs.

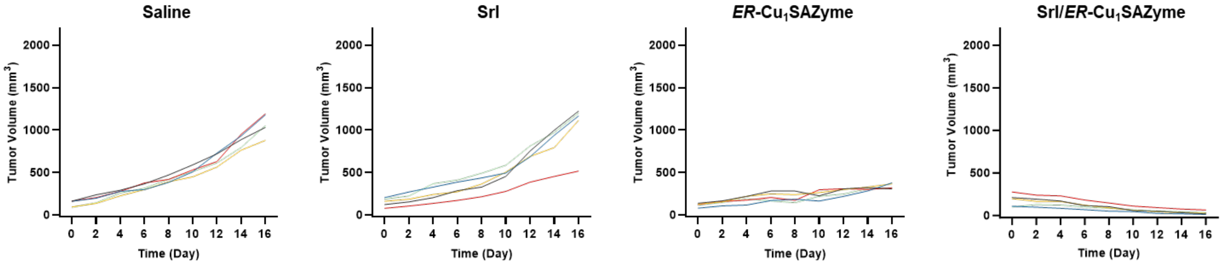

Figure S49. Tumor growth curves of individual mice in each group.

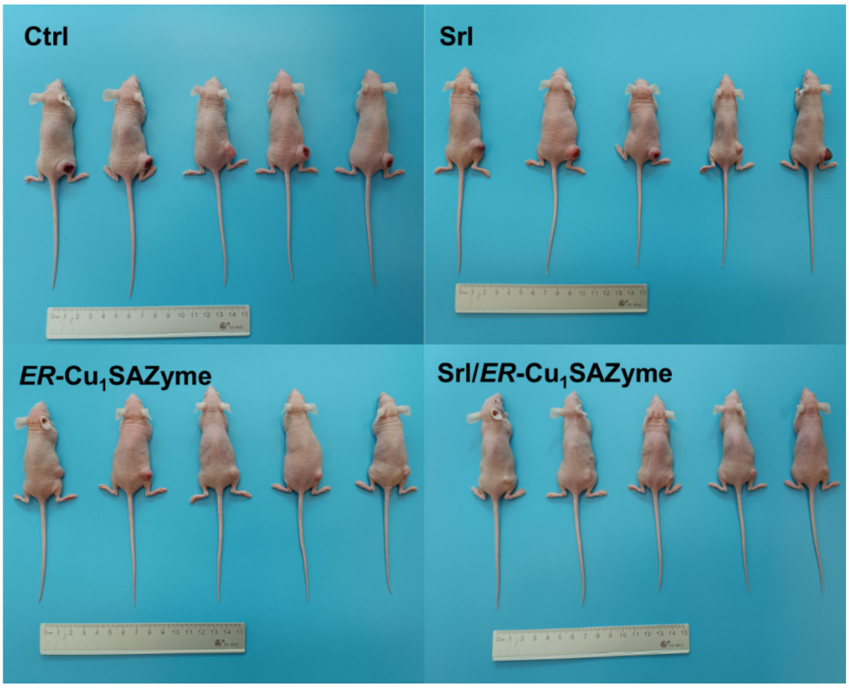

Figure S50. The photographs of mice 16 days after treatment.

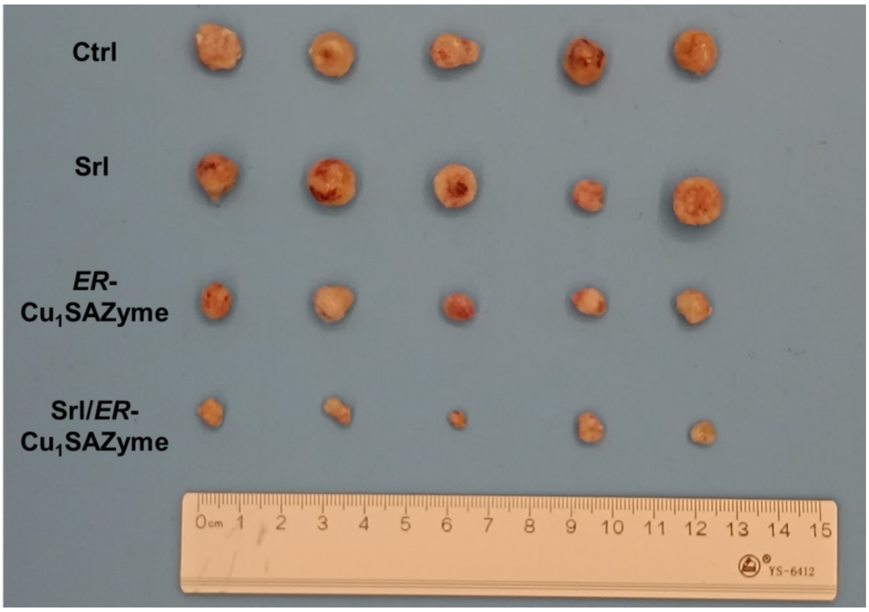

Figure S51. The photographs of xenograft tumors excised from mice 16 days after treatment.

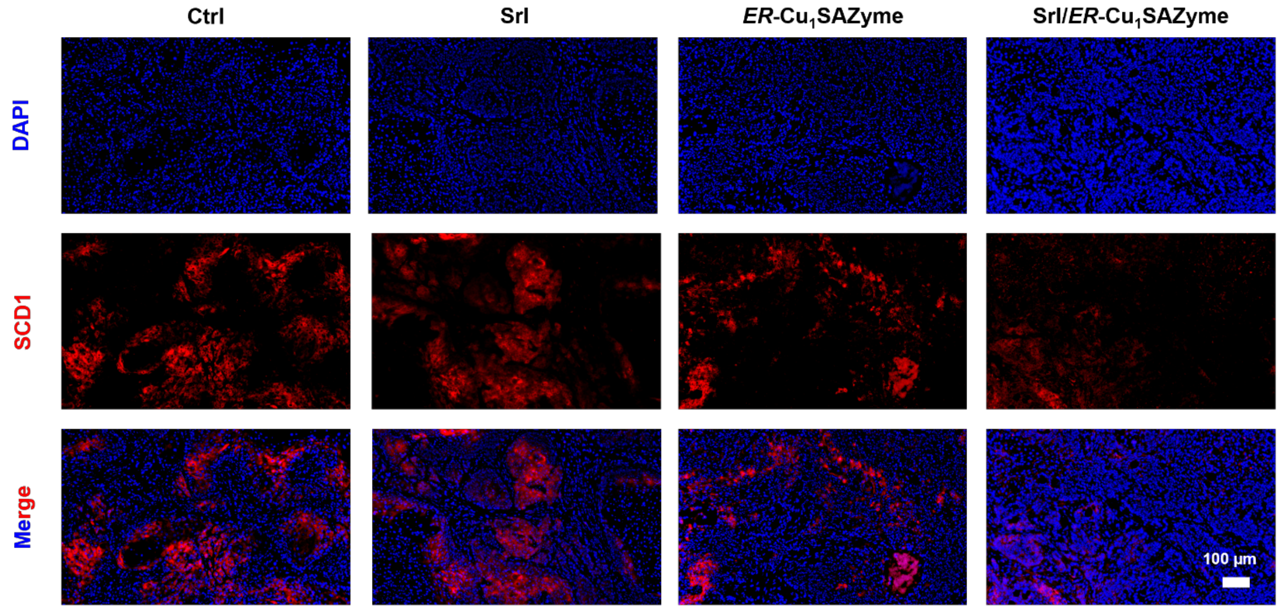

Figure S52. Histological microscopic images. The dissected tumors were stained with SCD1.

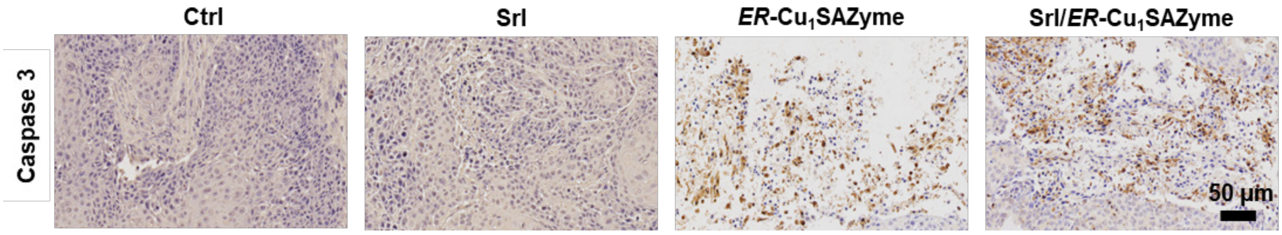

Figure S53. Histological microscopic images of dissected tumors stained with Caspase-3.

Supplementary Tables

Table S1. Cu concentration of *ER*-Cu_1SAZyme acquired from ICP, EDS and XPS measurements.

**Measurement**	**Cu Content (Mass %)**
ICP (bulk)	0.60
EDS (bulk)	0.73
XPS (surface)	1.15

Table S2. EXAFS data fitting results of Samples. Structural parameters of samples obtained by EXAFS fitting. There are the average coordination number (CN), path distance (R), Debye-Waller factor (σ^2), threshold energy correction (ΔE), and the R-Factor of the fitting. The S_0^2 used in the EXAFS fitting was estimated to be 0.97 based on the fitting of standard CuPc.

**Sample**	**Shell**	**CN**	**R(Å)**	**σ^2 (Å^2)**	ΔE(eV)	R-Factor
CuPc	Cu-N	4	1.94	0.0124	3.09	0.002
ER-Cu_1SAzyme	Cu-N	3.12	1.92	0.0180	3.09	0.003

Table S3. Molecular docking energy score. The binding energy score from molecular docking is generally considered to indicate a relatively strong interaction when it is less than -5 kcal/mol. Srl represents rapamycin.[4]

Protein	Ligand	Binding energy (kcal/mol)
mTOR	Srl	-8.442
SREBP1	Srl	-7.243
SCD1	Srl	-8.235

Table S4. Interaction residues between Srl and mTOR.

Name	Category
mTOR:LYS2218:NZ - Srl:O6	Electrostatic
mTOR: GLN1627: HE21- Srl: O2	Hydrogen Bond
Srl:H29 - mTOR:GLN1424:OE1	Carbon Hydrogen Bond
Srl:H88 - mTOR:GLN1424:OE1	Carbon Hydrogen Bond
Srl:C54 – mTOR: CYS1660	Hydrophobic
Srl:C24 – mTOR: ARG2217	Hydrophobic
mTOR: PRO1426 - Srl	Hydrophobic
mTOR: ARG2217 - Srl	Hydrophobic

Table S5. Interaction residues between Srl and SREBP1.

Name	Category
SREBP1:ARG889:HH12 - Srl:O13	Hydrogen Bond
SREBP1:ARG1126:HH11 - Srl:O13	Hydrogen Bond
SREBP1:ALA933 - Srl:C63	Hydrophobic
SREBP1:PRO991 - Srl	Hydrophobic
Srl:C54 - SREBP1:ARG986	Hydrophobic

Table S6. Interaction residues between Srl and SCD1.

Name	Category
SCD1:ARG347:HH21 - Srl:O13	Hydrogen Bond
Srl:C54 - SCD1:PRO57	Hydrophobic
Srl:C63 - SCD1:ILE54	Hydrophobic
SCD1:TYR41 - Srl:C24	Hydrophobic
SCD1:TYR306 - Srl:C50	Hydrophobic

Table S7. Clinical data of experimental cases.

Part	Number of patients	Male	Female	Average age
Tongue	12	7	5	63
Cheek	10	6	4	72.9
Inferior gingiva	3	3	0	75.3
Superior gingiva	3	1	2	65
Floor of the mouth	2	2	0	56
Total	30	19	11	65.17

Table S8. Primer sequence of qPCR.

Gene name	Primer name	Sequence
SCD	SCD_qPCR_F	GCTGTGGGTGAGGGCTTCC
	SCD_qPCR_R	AATGAAGAATGTGGTGAAGTTGATGTG
FASN	FASN_qPCR_F	CAGCGGCAAGCGTGTGATG
	FASN_qPCR_R	CCTCCTCCAGCGTCCAGTTG
FADS2	FADS2_qPCR_F	GAAGACGGCTGAGGACATGAACC
	FADS2_qPCR_R	CAATGCTCTCCAGGGCGATGATG
ACSL4	ACSL4_qPCR_F	GCTCTGTCACACACTTCGACTCAC
	ACSL4_qPCR_R	TTCCCTGGTCCCAAGGCTGTC
